# Melanoma-patient-derived xenograft multi-omics resource melPDomiX maps gain- and loss-of-function alterations

**DOI:** 10.1016/j.celrep.2026.117455

**Published:** 2026-06-03

**Authors:** Konstantinos Tsingas, Monzy Thomas, McKenna Reale, Min Xiao, Jayamanna Wickramasinghe, Yeqing Chen, Bryant Duong, Anastasia Lucas, Gatha Thacker, Haiyin Li, Haiwei Mou, Eric Ramirez-Salazar, Abdurrahman Elbasir, Jessie Villanueva, Xiaowei Xu, Ahron Flowers, Giorgos C. Karakousis, John T. Miura, Tara C. Mitchell, Ravi K. Amaravadi, Lynn M. Schuchter, Shujing Liu, Qi Long, Dave S.B. Hoon, Romela I. Ramos, Jose J. Catbagan, Matias A. Bustos, Jeffrey E. Gershenwald, Julie M. Simon, Jennifer A. Wargo, Michael A. Davies, Yiling Lu, Gordon B. Mills, Sonia Cohen, Aleigha Lawless, Tatyana Sharova, Dennie T. Frederick, Keith T. Flaherty, Nir Hacohen, Genevieve M. Boland, Noam Auslander, Meenhard Herlyn

**Affiliations:** 1 Molecular and Cellular Oncogenesis Program, Melanoma Research Center, The Wistar Institute, Philadelphia, Philadelphia, PA 19104, USA; 2 University of Pennsylvania, Philadelphia, PA 19104, USA; 3 Saint John’s Cancer Institute PHS, Depts Translational Molecular Medicine and Sequencing Center, Santa Monica, CA 90404, USA; 4 Department of Surgical Oncology, The University of Texas MD Anderson Cancer Center, Houston, TX 77030, USA; 5 Department of Melanoma Medical Oncology, Division of Cancer Medicine, The University of Texas MD Anderson Cancer Center, Houston, TX 77030, USA; 6 Oregon Health Science Center, Portland, OR 97239, USA; 7 Cancer Center, Mass General Research Institute, Harvard University, Boston, MA 02114, USA; 8 These authors contributed equally; 9 Senior author; 10 Lead contact

## Abstract

This study establishes melPDomiX, a multi-omics resource of melanoma-patient-derived xenografts. By integrating genomic, transcriptomic, and proteomic datasets through factor analysis and structural modeling, the authors distinguish gain- from loss-of-function variants. This resource enables investigation of treatment-refractory melanoma mechanisms using experimentally tractable tumor models for functional studies.

## SUMMARY

Melanoma is a common and aggressive cancer, with rising incidence in most developed countries. Major discoveries in melanoma biology have been rapidly translated, allowing cures for patients in late-stage disease. Despite these advances, many tumors remain refractory, in part due to an incomplete understanding of the genes and pathways gained or lost during melanoma tumorigenesis. To address this gap and provide a broadly useful resource for the scientific community, we established melPDomiX, a multi-omics cohort of melanoma-patient-derived xenografts. By linking mutations with transcriptomic and proteomic features, melPDomiX enables systematic characterization of gain- and loss-of-function alterations in treatment-refractory melanoma. Using multi-omics integration and structural-context representation, we demonstrate how this resource distinguishes gain- from loss-of-function variants and uncovers new candidate melanoma drivers and therapeutic targets. Together, melPDomiX provides a comprehensive, deeply profiled set of tumor models that supports mechanistic discovery and facilitates the development of improved treatments for this devastating heterogeneous malignancy.

## Graphical Abstract

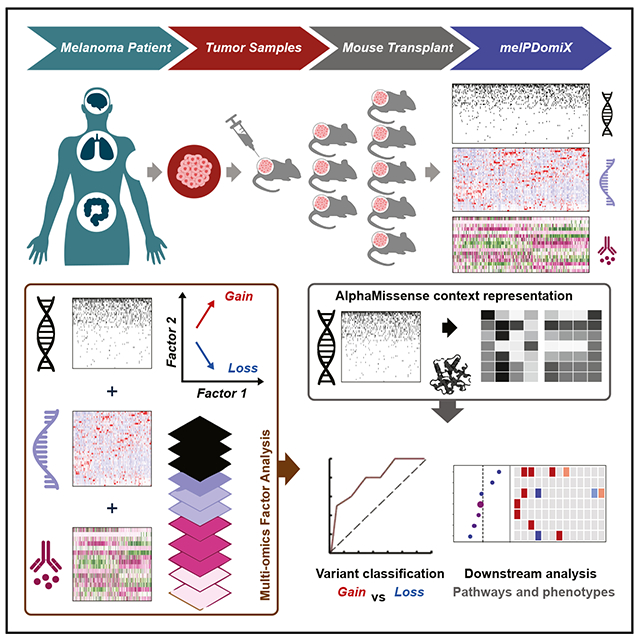

## INTRODUCTION

Melanoma is among the most heterogeneous and highly mutated cancer types,^1,2^ with a rapidly rising global burden over the recent years.^3,4^ Through genetic drivers and mutagenesis resulting from exposure to ultraviolet (UV) radiation, melanoma is associated with heterogeneous pathogenesis that depends on various factors including geography and racial identity.^5,6^ Interacting intrinsic and extrinsic elements, such as genetic susceptibility to UV radiation and UV-damage-induced mutagenesis,^7,8^ drive immune suppression in the skin^9,10^ and modulate melanoma initiation, progression, and outcomes. Melanoma poses major challenges for precision medicine and the development of new therapeutics due to its multiple genetic drivers and extreme intra- and inter-tumor variability.^11^ Advanced cutaneous melanoma has been associated with poor outcomes and low survival rates.^8,12^ However, recent progress in immunotherapy and targeted therapies have revolutionized the treatment of melanoma, allowing for improved survival rates and even cures at late stages of the disease.^13^

Recent therapeutic advancements have been largely enabled by new insights into the molecular and genetic basis of melanoma, as well as the tumor microenvironment. Characterization of *BRAF* V600 gain-of-function (GOF) in approximately 50% of melanomas drove the development of highly potent BRAF inhibitors (BRAFis).^14,15^ A subsequent discovery of acquired resistance through activation of alternative signaling pathways led to the highly efficacious BRAFi/MEK inhibitor (MEKi) combination.^16,17^ Beyond these two examples, there are no other targeted therapy approaches that produce durable responses in melanoma, despite the cataloging of thousands of mutations and potential targets in efforts such as The Cancer Genome Atlas (TCGA) Genomic Classification of Cutaneous Melanoma.^18^ The discovery of immune checkpoints, which regulate the immune system in cancer,^19,20^ allowed for subsequent development of antibodies that block immune checkpoints and reinvigorate exhausted T cells to attack cancer cells. The efficacy of these antibodies was first shown in treating cutaneous metastatic melanomas.^21,22^ While immune checkpoint inhibitors have made a major impact, many patients are resistant to immunotherapy,^21,23,24^ and there is a need for developing new rational therapies for immunotherapy-resistant melanoma.

These major discoveries and clinical advances for melanoma were made possible through the characterization of protein GOF and loss-of-function (LOF) that occur during disease development and as resistance emerges. To allow the design of new therapeutic strategies for patients who are resistant to existing therapies,^24,25^ it is crucial to first distinguish GOF and LOF drivers of melanoma through genetic and molecular alterations. These insights and the availability of fully characterized pre-clinical melanoma models can lead to new therapeutic approaches targeting additional melanoma processes and resistance mechanisms.

In this study, we established melPDomiX, a unique cohort of melanoma-patient-derived xenograft (PDX) models, profiled with whole-exome sequencing, RNA sequencing (RNA-seq), and reverse phase protein array (RPPA). In contrast to TCGA skin cutaneous melanoma (SKCM cohort),^18^ melPDomiX represents primarily advanced melanomas from patients treated with targeted and/or immunotherapies. We utilized this cohort to characterize GOF and LOF mutations in melanoma. We found GOF mutations by identifying selective pressure at specific residues. Integrating multi-omics features allowed a clear separation of GOF and LOF mutations. Finally, incorporating structural context enabled the prediction of new candidate GOF melanoma variants, including those in the *KANSL1* and *ERBB3* genes. This study improves our understanding of the molecular, pathway, and structural basis of GOF and LOF drivers of melanoma, providing a foundation for developing novel therapies for advanced disease.

## RESULTS

### PDX models reflect the genetic, molecular, and therapeutic heterogeneity of clinical melanoma

**Figure 1.**
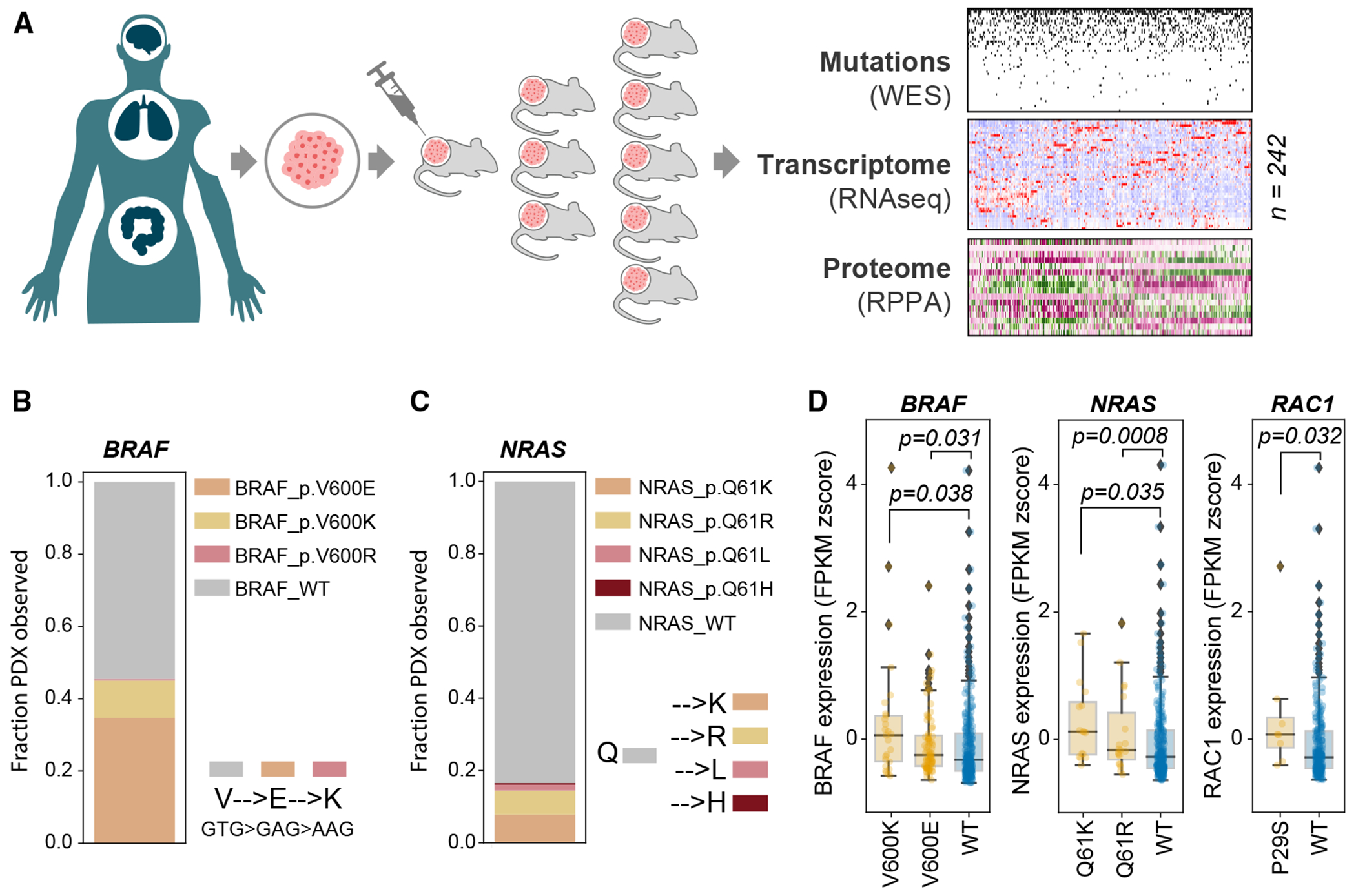
The melPDomiX cohort (A) Schematic overview of the PDX generation and omics collection pipeline. (B and C) The fraction of the different types of *BRAF* and *NRAS* substitutions observed in *BRAF* V600 and *NRAS* Q61 across the melPDomiX cohort. The amino acid, substitutions, and codon change are specified. (D) Boxplots showing *BRAF, NRAS*, and *RAC1* expression levels for hotspot *BRAF, NRAS*, and *RAC1* melanoma mutations. One-sided Wilcoxon rank-sum test unadjusted *p* values are reported.

To establish the melanoma PDX multi-omics cohort (melPDomiX), we have genomically and molecularly characterized 242 melanoma samples with PDXs from eight institutions (Table S1). PDXs were generated from these samples as previously described.^26^ In this study, 242 tumor samples were assessed by whole-exome sequencing (WES), RNA-seq, and RPPA for the first time and integrated with matched donor clinical data (Figure 1A). These fully characterized models have been made available for the broader scientific community. The vast majority of PDXs are from advanced stage melanomas, where 62% of the PDXs are from stage IV and 19% from stage III tumors, with 9.5% (23 tumors) from brain metastases. One hundred one PDXs are from patients treated with immune checkpoint inhibitors, and 60 are from patients treated with BRAFi/MEKi, all having response information available. Tumor subtype is characterized for 61% of the samples, where cutaneous is the most common subtype, followed by mucosal melanomas (Table S1). The best clinical response to immunotherapies (considering responses pre- and post-biopsy collection) was complete or partial response (CR/PR) for 13 PDXs, stable disease (SD) for 9, mixed response (MR) for 7, and progressive disease (PD) for 72 PDX tumors. Response (CR/PR) to BRAFi/MEKi was observed in 17 of the PDX tumors, while 11, 10, and 22 PDXs from patients exhibited SD, MR, and PD, respectively. Therefore, the melPDomiX cohort mostly represents advanced stage and treatment-resistant melanoma tumors, in contrast to TCGA SKCM.

BRAF inhibitors target the most frequent and clinically relevant somatic mutations in melanoma within the *BRAF* hotspot, the most common of which is *BRAF* V600. A single mutation in the V600 residue GTG→GAG results in the V600E variant, whereas a double substitution GTG→AAG results in V600K. In melanoma, many double substitutions arise from UV-induced photoproducts rather than independent events.^27^ Regardless of mechanism, recurrent codon changes indicate strong positive selection for oncogenic activation. In melPDomiX, 45% of PDXs are *BRAF* V600 mutated, with 35% of PDXs harboring the V600E mutation and 10% harboring the V600K mutation (Figure 1B), similar to previous reports.^18,26^ This indicates a strong selective pressure whereby specific residues are recurrently mutated across tumors, a pattern typically characterizing oncogenic cancer mutations.^28^ We therefore estimated selective pressure across residues of different genes and found a similar selective pressure in the *NRAS*-mutant melanomas, where multiple distinct substitutions in position Q61 give rise to different recurrent amino acid changes (Figure 1C).

Gene variants were linked with treatment benefit by employing an enrichment analysis (STAR Methods). We identified one mutation significantly associated with response and multiple mutations in a similar locus associated with non-response to BRAFi/MEKi. The R465Q mutation in the anaphase-promoting complex subunit 4 gene (*ANAPC4*) occurred in approximately 50% of PDXs with complete or partial response (CR/PR), mixed response (MR), or stable disease (SD), whereas none of the PDXs from patients with progressive disease (PD) after BRAFi/MEKi presented this mutation (Extended Data Figure 1A; Tables S2 and S3).

Therefore, *ANAPC4* R465Q status is a promising new candidate for predicting clinical benefit from BRAFi/MEKi treatment in melanoma (Fisher’s exact test false discovery rate [FDR] q < 0.1; Extended Data Figure S1A). To further evaluate this association, we first analyzed TCGA SKCM data, in which a subset of *BRAF* V600-mutant tumors similarly harbored the germline *ANAPC4* R465Q variant (Extended Data Figure S1B). Because TCGA lacks clinical response data for BRAF/MEK inhibitor therapy, we examined progression-free survival as a surrogate outcome. *ANAPC4* R465Q-mutant tumors exhibited a trend toward improved progression-free interval compared with *ANAPC4* wild-type tumors (log rank *p* = 0.086; Extended Data Figure S1C). Due to the absence of melanoma cohorts with paired germline sequencing and annotated clinical responses to BRAF/MEK inhibition, we next assessed drug responsiveness *in vivo*. Two *ANAPC4* R465Q-mutant melanoma models and one *ANAPC4* wild-type model were implanted into NSG mice and treated with BRAF/MEK inhibitors, with tumor growth monitored longitudinally. Consistent with the melPDomiX and TCGA analysis, *ANAPC4* R465Q-mutant tumors showed enhanced sensitivity to BRAF/MEK inhibition, as evidenced by reduced tumor growth during treatment (Extended Data Figure S1D).

Interestingly, BRAF inhibition was recently shown to alleviate cartilage degradation,^29^ and *ANAPC4* mutations have been linked to osteoarthritis^30^ (STAR Methods). Four highly co-occurring likely haplotype germline variants (G40V/*, I94L, and E86D) in class II major histocompatibility complex (MHC) gene *HLA-DPB1* were significantly associated with poor response to BRAFi/MEKi treatment (Extended Data Figure S1A; Tables S2 and S3, STAR Methods).

A main goal of this study is to utilize the melPDomiX cohort to identify features that characterize GOF and LOF mutations in melanoma. To this end, we first curated a list of literature-reported GOF and LOF melanoma variants (Table S4). Given that only a limited number of variants have been characterized as GOF or LOF, we explored recurrent substitution, gene and protein expression changes, and structural context patterns that may underlie the functional effects of mutations. To assess our ability to predict GOF or LOF based on molecular features, we evaluated the agreement between GOF- and LOF-classified variants when using various molecular attributes and when comparing to literature-reported GOF/LOF annotations for consistency.

Recurrently mutated residues and the presence of multiple distinct substitutions at the same protein position indicate a strong selective pressure, consistent with a requirement for oncogenic, GOF activity.^28^ This contrasts LOF or tumor suppressive mutations, which tend to be distributed throughout the gene and arise from diverse disruptive variants.^28^ We examined proteins with multiple recurrent substitutions at the same residue across our PDX cohort to identify potential new GOF candidates. We detected 678 such variants across 400 genes, where many occurred in long genes such as *TTN* and mucins, or high variability regions such as MHC, which tend to accumulate more mutations. In addition, we identified highly recurring multiple substitution variants in the leukocyte immunoglobulin-like receptor B1 gene, *LILRB1* Y99 position (Y→I/F/N), and melanoma-associated antigen C1 (*MAGEC1*) position P239 (P→S/R) (Table S5). However, recurrent multiple substitutions were largely absent from known melanoma driver genes, motivating further analysis to identify additional features distinguishing true GOF and LOF mutations from the numerous passenger variants present in melanoma.

The melPDomiX cohort allows us to evaluate how each variant may influence the gene or protein expression in melanomas (Tables S6 and S7). Starting with the best-studied GOF mutations in melanoma, *BRAF* V600 and *NRAS* Q61, we found that these variants exhibit increased gene expression levels of *BRAF* and *NRAS*, respectively (Figure 1D). We also observed increased gene expression levels in other well-characterized melanoma GOF hotspot variants, such as *RAC1* P29 (Figure 1D). In contrast, LOF mutations in melanoma, such as *TP53* truncating mutations, showed lower gene expression levels than wild type. Because rare variants are often underpowered for conventional hypothesis testing due to limited sample size, we systematically characterized the expression fold change in the presence of each variant to establish a collection of potentially expression increasing and decreasing variants in melanoma.

### Identification of melanoma variants associated with gain or loss of expression

We evaluated how the presence of different variants observed in the PDX cohort influenced mRNA expression levels of each respective gene. To confirm the patterns found using the PDX cohort, we additionally utilized TCGA SKCM^18^ cohort of somatic or germline mutations and RNA-seq data. Variant-gene expression changes observed in our PDX cohort that were confirmed in TCGA SKCM dataset were extracted into a final collection of melanoma gain- or loss-of-expression variants (GOE and LOE, respectively; Table S8).

Interestingly, we observe 86% agreement between potential GOE variants with literature-reported GOF variants or LOE variants with confirmed LOF variants (Table S8). In melanoma, all literature-reported GOF variants showed a consistent increase in gene expression, even though GOF may relate to change in the protein biochemical activity or stability and not necessarily expression itself. Beyond melanoma, we analyzed previously annotated pan-cancer driver genes^31^ and observed a similar trend, where mutations in tumor suppressor genes were generally associated with lower expression levels, whereas mutations in oncogenes were associated with higher expression levels (Extended Data Figure S2A). Overall, this indicates that variant-observed expression changes could be used to prioritize variants for downstream GOF and LOF characterization or as a proxy for functional classification in melanoma.

**Figure 2.**
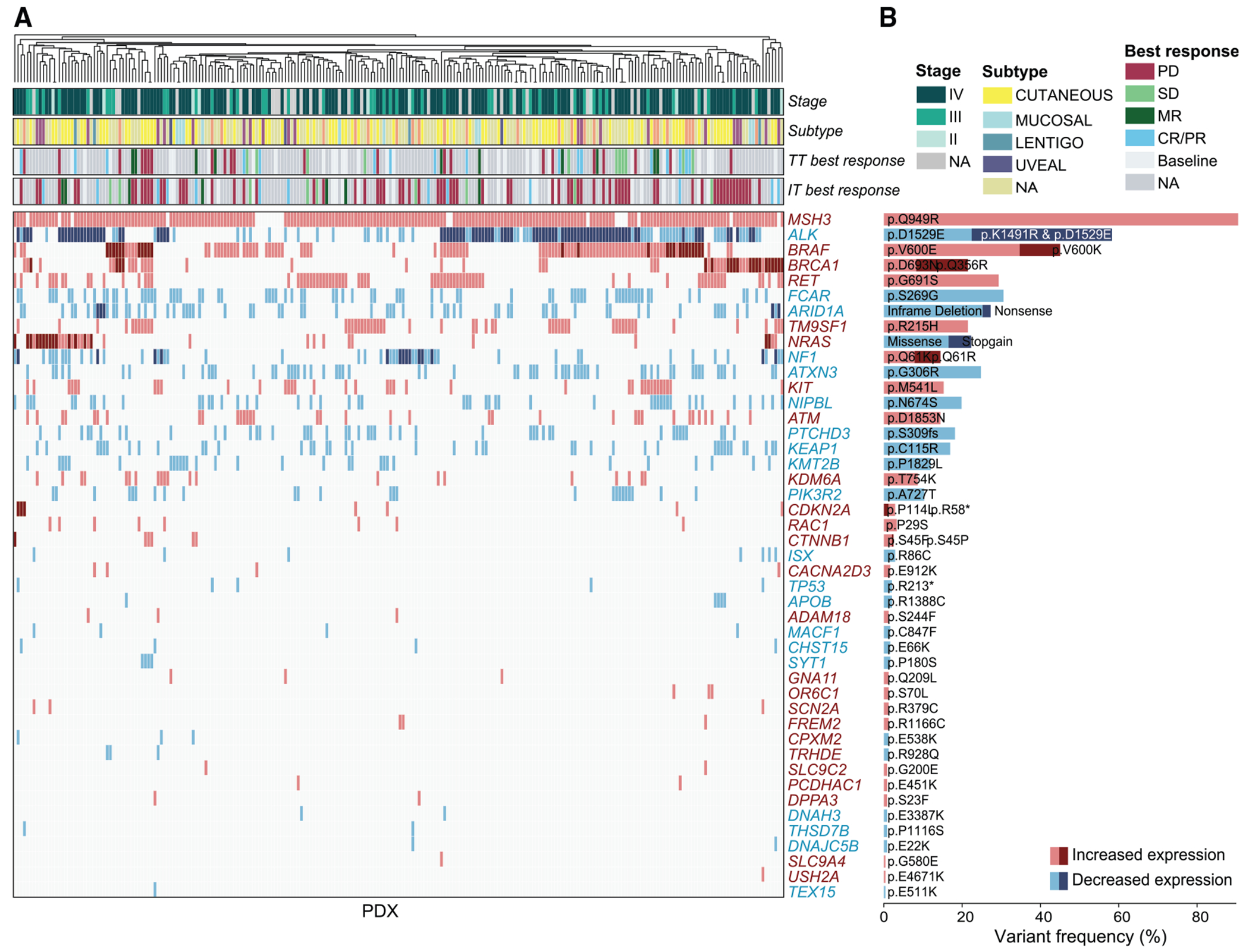
Potential gain- and loss-of-expression melanoma variants (A) Lower panel: the mutational map of variants with observed increased or decreased levels of the respective gene (RNA-seq) in the PDX cohort. All selected variants are identified through the PDX cohort and verified in TCGA SKCM cohort. Upper panels: clinical information of PDX models, including melanoma stage, subtype, and targeted therapy (TT) or immunotherapy (IT) best response observed in the patient. (B) Variant frequency (%) of the GOE and LOE variants classified by change of expression across the PDX cohort. Reds represent occurrences of expression-based GOE, and blues represent occurrences of expression-based LOE variants (light colors are the most frequently occurring substitution, and darker colors represent the less frequent substitution of the same residue).

We systematically investigated this pattern to identify common GOE and LOE melanoma variants. The most common GOE variant was Q949R in the *MSH3* mismatch repair complex gene (corresponding to SNP rs184967; Figure 2), which was observed only in germline. While *MSH3* Q949R has been classified as a common benign variant, it presents with a consistent increase of *MSH3* expression in both melPDomiX and TCGA SKCM. Additionally, it had been reported as variant of unknown significance for Lynch syndrome^32^ and has been significantly associated with colorectal cancer risk.^33^ *MSH3* Q949R is therefore a potentially interesting variant for testing specifically in melanoma. The second and third most common GOE variants were *BRAF* V600, which occurred somatically, and *RET* G691S,^34^ which occurred both somatically or in germline (Table S8). Additionally, we identified several newly reported GOE candidates including *TM9SF1* R215H, *CTNNB1* S45, and *ATM* D1853N. *CTNNB1* p.S45 variants are classified as a likely pathogenic mutation for melanoma and other carcinomas in ClinVar and are reported to be GOF. For the latter *ATM* D1853 variant, we observed two different variants (D→N/V) among the melPDomiX cohort, supporting its predicted GOE role in melanoma. Interestingly, *ATM* D1853N variant was associated with patients with multiple primary melanomas^35^ and melanoma risk.^36^

The most frequently observed expression-based, likely LOE variant was in the *ALK* gene, observed in 58% of PDXs, with two co-occurring germline variants observed in our PDX cohort, K1491R and D1529E, such that all samples with K1491R mutations also had D1529E mutations. We observed lower *ALK* expression in the presence of these co-occurring mutations. We also observed lower expression of the *NF1* tumor suppressor gene in the presence of missense and deactivating stop gain mutations in *NF1*, as expected (Figure 2). Additional common expressionbased potential LOE variants identified through this analysis were *KEAP1* C1115R,^37^ *FCAR* S269G, and *NIPBL* N674S.

Somatic and germline status of each variant was determined by examining mutations called with and without matched patient blood cells in melPDomiX. In contrast to sample-specific variant somatic or germline status, which may be inferred precisely in the presence of matched blood sequencing, cumulative classification of each variant into a likely somatic or germline status was based on combined evidence from all melPDomiX samples as well as TCGA SKCM (Table S8). The majority of likely GOE variants in melPDomiX were somatic mutations (71%, hypergeometric enrichment; *p* = 0.04). In contrast, likely LOE variants showing lower gene expression tended to be germline mutations (58%, hypergeometric enrichment; *p* = 0.02). We verified the trend toward GOE variants occurring somatically and LOE variants occurring in germline in TCGA SKCM samples (Table S8). Further, we verified that a similar association was observed for the literature-reported GOF variants (hypergeometric enrichment; *p* = 0.005; Tables S4, S5, S6, S7, and S8). The results suggested a new link between somatic variants with GOF and GOE and between germline variants with LOF and LOE. Despite this strong overall association, not every variant followed this trend. Most of the germline variants that were annotated as GOE were also observed somatically in a smaller subset of samples. For example, *KIT* M541L and *TM9SF1* R215H were observed mostly as germline variants but also occurred somatically in multiple PDXs (Tables S2 and S3). Therefore, almost all the likely GOE variants can occur somatically in melanoma. In contrast, GOE somatic variants were almost always never mutated in germline, indicating that many oncogenic germline mutations may not be tolerated.

**Figure 3.**
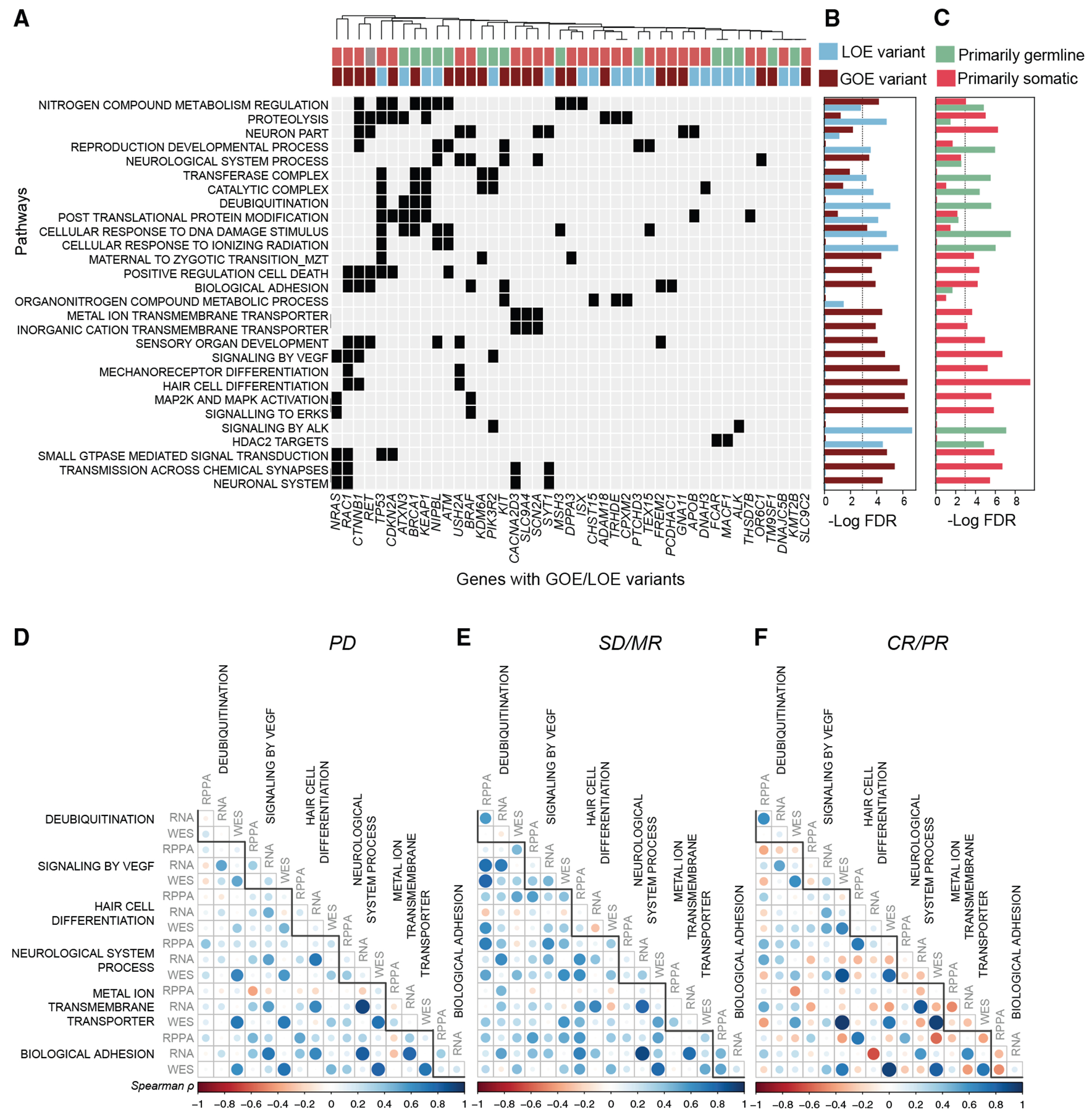
Pathways and treatment response associations of potential GOE and LOE variants in melanoma (A) Pathways enriched with the annotated GOE and LOE variants by association with increased or decreased gene expression levels. The genes belonging to each pathway are specified, and the GOE/LOE and somatic/germline classifications of each variant for these genes are noted. (B and C) The negative log-transformed, FDR-corrected enrichment *p* value for each pathway, for pathway enriched for GOE and LOE (B) or somatic and germline variants (C). A threshold of 0.05 is marked with a dashed line. (D–F) Correlation plots showing pathway scores calculated using each omics type, for PDXs from patients demonstrating progressive disease (PD) (D), stable disease/mixed response (SD/MR) (E), and complete/partial response (CR/PR) (F) to immunotherapy.

To investigate and link the gained and lost biological processes and signaling pathways in melanoma with somatically and germline occurring mutations, we performed a pathway enrichment analysis (Figure 3A). We explored pathways enriched with our list of candidate GOE and LOE variants, harnessing pathway annotations from five different sources (Figure 3B). Additionally, we performed pathway enrichment analysis on genes with mutated variants occurring somatically and in the germline through this set (Figure 3C). Similar to the overall variant trends, we observed enrichment of overlapping pathways among genes harboring GOE variants and somatically mutated variants. Likewise, similar pathways were enriched among genes with LOE variants and those carrying germline mutations (Figures 3B and 3C). For instance, we found that vascular endothelial growth factor (VEGF) signaling is significantly associated with GOE, consistent with the known role of angiogenesis in melanoma,^38^ and VEGF was also uniquely associated with somatically mutated genes. Similarly, mitogen-activated protein kinase (MAPK) activation and ERK signaling were associated with LOE and somatic mutations.^39,40^

We next examined how GOE and LOE pathways may be regulated and how this pathway regulation correlates with clinical outcomes. To this end, we derived a PDX sample-level pathway score for each omics type, evaluating mutations, as well as gene and protein expression levels within each of the enriched pathways (see STAR Methods for details). We explored pathway-level regulation by linking the mutational pathway scores to the RNA-seq and RPPA pathway scores across melPDomiX from patients who were immunotherapy responders and non-responders. Importantly, we identified multiple pathways in which mutations positively regulate gene or protein expression in non-responders to immunotherapy or in patients with mixed responses. Most of those are expression-based GOE pathways, in which a tight positive regulation is expected. However, mutations in these pathways negatively regulate gene or protein expression in immunotherapy responders. Such pathways include biological adhesion, neurological system processes, and metal ion transmembrane transporter activity (Figures 3D–3F). The negative regulation of mutants in these GOE pathways by expression may therefore inform melanoma immunotherapy responses, distinguishing complete and partially responding tumors. In contrast, we did not identify pathways in which positive regulation was associated with response to immunotherapy. Interestingly, we observed similar, albeit milder, associations when evaluated according to melanoma targeted therapy response (Extended Data Figure S2B), indicating a potential general link between negative regulation of GOE pathways and treatment responses in melanoma.

We additionally explored co-occurrence and mutual exclusivity patterns of the identified expression-based GOE and LOE variants. GOE variants were more commonly mutually exclusive with each other, such as *BRAF* V600E, which was mutually exclusive with *NRAS* Q61 and *RAC1* mutations. In contrast, *NRAS* Q61 and *RAC1* mutations showed co-occurrence with LOE variants, as is the case for *KEAP1* with *NRAS* and *RAC1* mutations (Extended Data Figure S2C).

### Uncovering the functional role of melanoma variants through multi-omics integration

**Figure 4.**
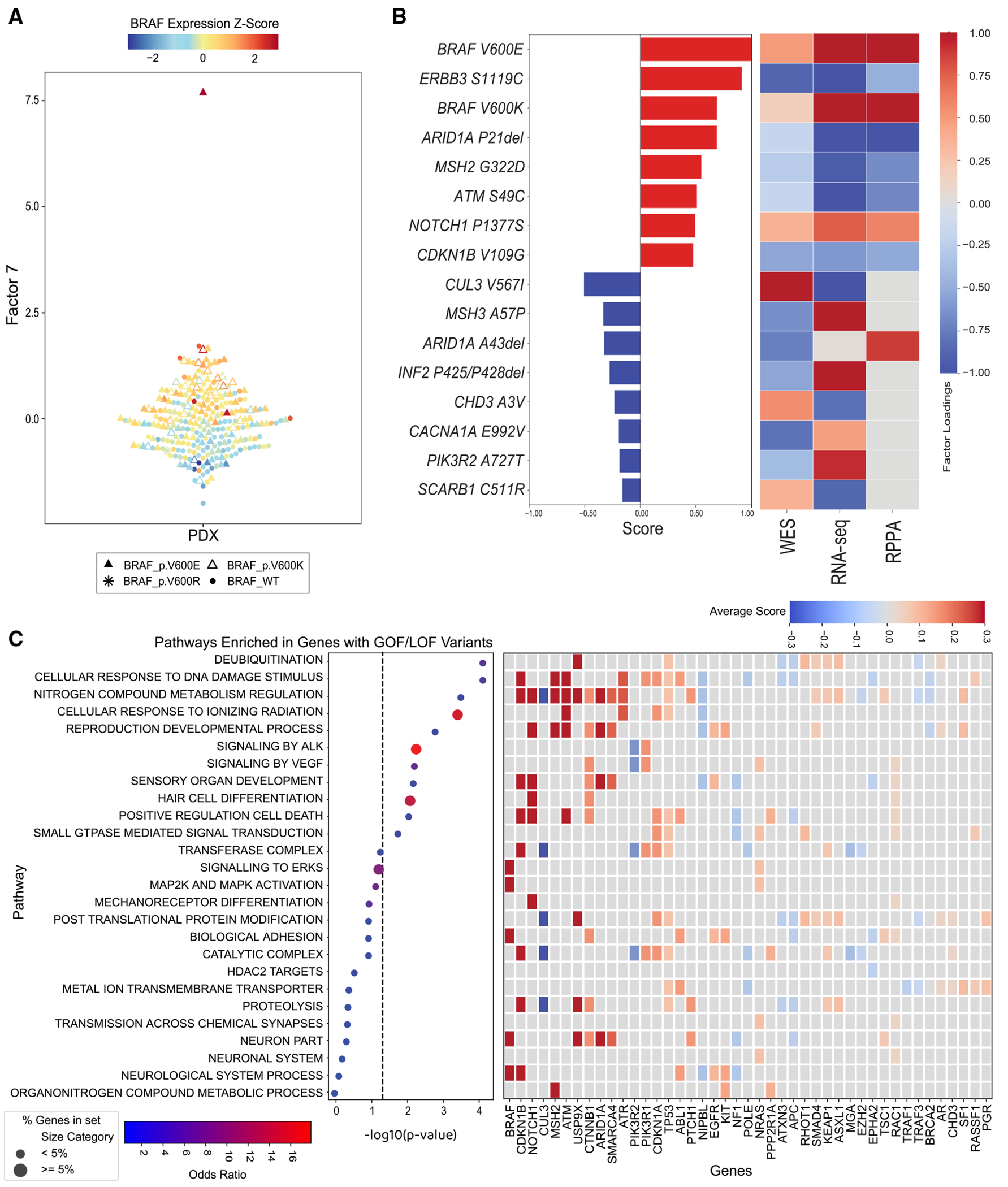
Multi-omics integration uncovers patterns of potential LOF/LOE and GOF/GOE variants (A) Swarm plot overlaying the distribution of latent factor 7 across PDXs with variant status and normalized *BRAF* gene expression values. PDXs with positive factor 7 values are shown to have larger average gene expression and increased frequency of *BRAF* V600 variants. (B) Barplot showing the highest ranking GOE and LOE variants by weighted score calculated from latent interaction across omics, and heatmap showing the corresponding raw factor loadings for each omics type. The factor chosen to represent final GOE/LOE status is determined from the maximum observed weighted score across all factors. The prediction score was calculated by evaluating the direction of contribution of variants, genes, and proteins. (C) Left panel: dot plot showing odds ratios and negative log-transformed FDR-corrected *p* values from pathway enrichment for genes with likely GOF/GOE or LOF/LOE variants found by multi-omics annotation. A threshold of 0.05 is marked with a dashed line. Right panel: heatmap showing the average likely GOF/LOF annotation score across variants for each gene overlapping with included pathways.

Integrating heterogeneous multi-omics data derived from the melPDomiX cohort enables a comprehensive understanding of joint mechanisms underlying melanoma. By combining WES, RNA-seq, and RPPA data, we employed multi-omics factor analysis (MOFA)^41^ to build a latent embedding that captures coordinated variation across omics layers. GOF and GOE variants such as *BRAF* V600E and V600K were consistently weighted positively across factors, coinciding with increased gene and protein expression (Figure 4A). Conversely, LOF/LOE variants showed opposite patterns, such as *KMT2B*, *PIK3R2*, and *TP53* (Extended Data Figure S3B). The MOFA model estimated 14 latent factors, with factors 5–9 capturing mixed contributions across omics layers (Extended Data Figure S3D).

We therefore used multi-omics integration to identify latent factors representing biological mechanisms and to classify likely GOF/GOE and LOF/LOE variants through variant-specific scoring, uncovering functional links between variants and gene or protein expression that are not apparent from single-omics analyses. We leveraged the sign and magnitude of factor loadings across omics to classify variants. Specifically, concordant loadings of a variant and its gene/protein across factors indicate gain, while opposite signs suggest loss. Using this approach, *BRAF* V600 single and double substitutions were correctly classified as GOF, and the scoring also accurately classified other expression gain variants (Figure 4B, Extended Data Figure S3E). Literature-reported variants aligned with the composite score (receiver operating characteristic - area under the curve [ROC AUC] = 0.75, 95% bootstrapped confidence interval [CI]: [0.36, 0.93], and precision-recall curve AUC [PRC AUC] = 0.86, 95% bootstrapped CI: [0.65, 0.97]) (Extended Data Figure S4A), although the confidence interval for the ROC AUC reflects increased uncertainty given the imbalanced number of true GOF/LOF variants. Furthermore, applying the same framework to TCGA SKCM data enabled separation of GOF vs. LOF and GOE vs. LOE variants, pinpointing additional well-known GOF and LOF melanoma variants (Extended Data Figure S6). Pathway enrichment analysis of predicted GOF/GOE or LOF/LOE variants revealed association with programmed cell death, involving genes such as *NOTCH1*, *ATM, CDKN1B*, and *CTNNB1*, with most enriched pathways overlapping GOE variants overall (Figure 4C). The *NOTCH1* GOF/GOE was driven solely by the P1377S variant, suggesting a potential activating role in regulatory pathways. Highly ranked variants not captured in the pathway analysis included *ERBB3* S1119C, the second highest scored GOF/GOE variant after *BRAF* V600E (Figure 4C).

### Structural context of expression or function gain and loss variants in melanoma

GOF and LOF are disruptive variants.^42,43^ To explore how protein structure features are linked with GOF vs. LOF variants, we utilized AlphaMissense structural context variant representation.^43^

**Figure 5.**
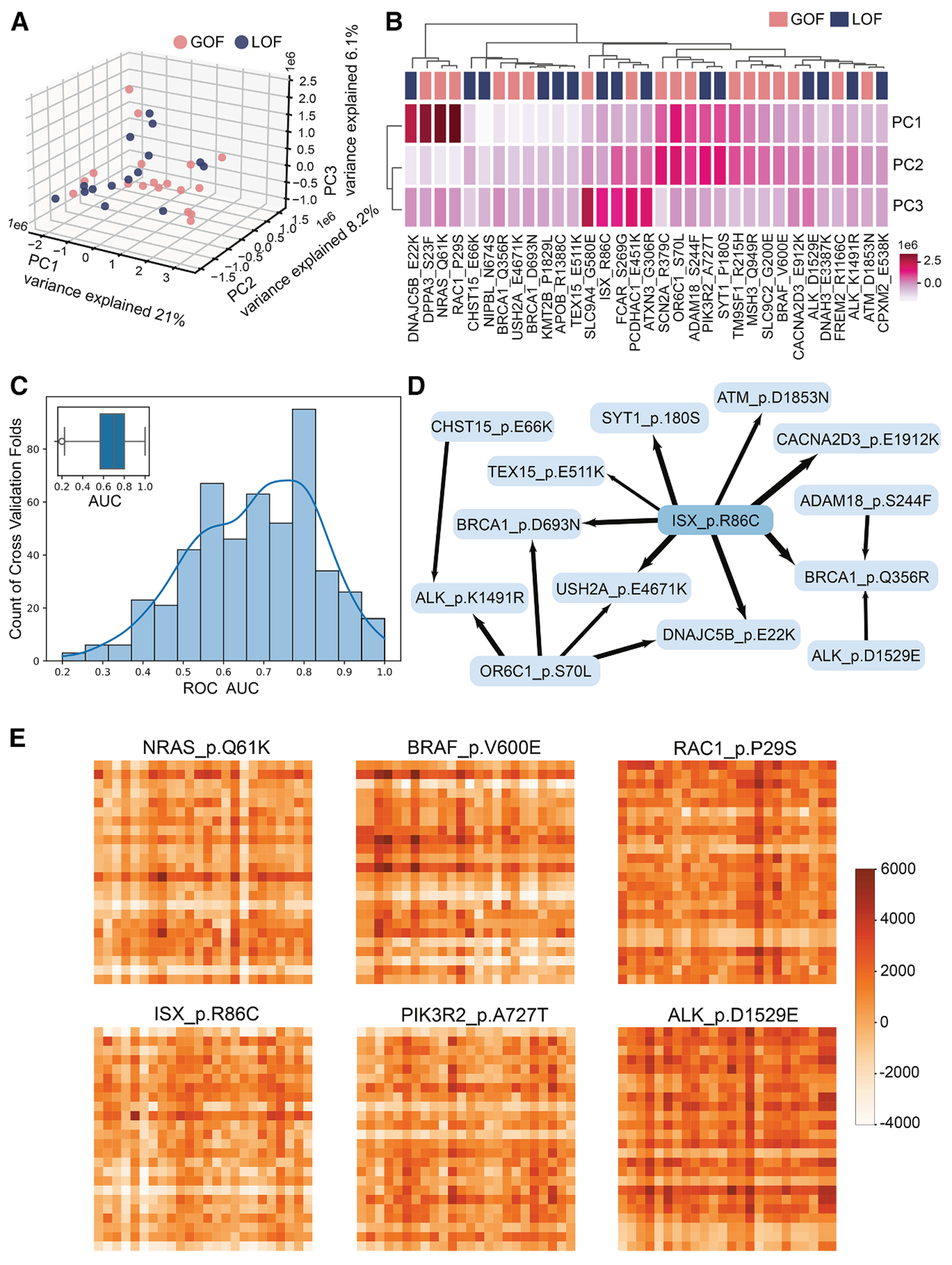
Exploratory structural context characterization of melanoma GOE and LOE variants (A) The first three principal components of the default AlphaMissense pair representations explain 35% of the variance. (B) Heatmap showing the PCA loadings assigned to the first three principal components and GOE or LOE variants clustered hierarchically. (C) Performance distribution using the area under the receiver operating characteristic curve for GOE/LOE prediction using K-nearest neighbors classifiers through 500 different random folds. The first 12 PCs were used for classification, explaining in aggregate approximately 71% of the variance. (D) Graph representation of variant contribution to GOE/LOE prediction of other variants. *ISX* R86C is the strong predictor for other variants (edge weight >0.7). (E) 25 × 25 regions of heaviest PCA loadings along the first dimension of the default 256 × 256 × 128 AlphaMissense representation.

We first applied AlphaMissense^43^ to extract representations of the GOE and LOE variants (Table S8). The principal components of GOE variants from the AlphaMissense embedding formed distinct clusters, separating GOE from LOE variants based on the reduced dimension context representation (Figure 5A). For example, *NRAS, RAC1*, and *DPPA3* variants formed one cluster, whereas *BRAF* V600 was clustered with *MSH3, SLC9C2*, and GOF *TM9SF1* variants (Figure 5B). K-nearest neighbors classifiers trained to distinguish GOE from LOE variants achieved an average ROC AUC of 0.7, supporting that structural information representation can inform the GOE vs. LOE context of melanoma variants (Figure 5C). *ISX* R86C was the strongest contribution to correct prediction (Figure 5D).

Importantly, the same approach applied to literature-reported GOF and LOF variants showed similarly strong performance (Extended Data Figures S4B–S4D). These results demonstrate that structural context can distinguish melanoma-specific GOF from LOF missense variants (Table S9). Analysis of embedding dimensions provided further insight into amino acid context patterns of GOF vs. LOF variants (Figure 5E).

Given that multi-omics integration and structural context representation inform GOE vs. LOE and GOF vs. LOF variants, we applied these methods to variants not identified by observed changes in mRNA expression levels. Variants most strongly predicted as GOF/GOE or LOF/LOE were classified using AlphaMissense embeddings and PCA (Extended Data Figure S5). Top predictions included four mutually co-occurring variants in the *KANSL1* genes (K104T, I1085T, S718P, and N225D) and *ERBB3* variant S1119C. Mutations in the *KANSL1* gene are causal for the Koolen-de Vries syndrome, where skin phenotypic abnormalities are common, and some rare cases of melanoma at an early age have been reported.^44^ *ERBB3* (HER3) mutations are considered GOF in most cancer types^45^ and have been implicated in melanoma.^46^ Also, the S1119C variant has been reported only in breast cancer and has been associated with poor outcomes.^47^

## DISCUSSION

Classification of variants into GOF and LOF has been critical for melanoma research and the development of more effective therapeutic strategies. For example, the characterization of *BRAF* V600 GOF mutants has led to the most effective targeted therapeutic strategy for metastatic melanomas, where additional variants are being explored at different clinical development stages.^48^ Understanding tumor biology by characterization of the effects of these mutations through genomics and other types of omics modalities has enabled recent clinical breakthroughs. In melanoma, such new knowledge has significantly improved survival outcomes for more than half of the patients with advanced disease, allowing for longer survivorship and even cures for patients who were previously considered to be without viable treatment options. These advances highlight the need for robust, well-characterized resources that can systematically evaluate functional variation, motivating the development of the melPDomiX cohort.

The melPDomiX cohort provides a deeply characterized dataset that includes WES, RNA-seq, and RPPA data paired with annotations from patient tumors. To exemplify the broadness of this dataset for functional interpretation of genomic alterations, we leveraged this resource to systematically characterize GOF and LOF variants in melanoma. Variants associated with increased or decreased gene or protein expression, named GOE and LOE variants, respectively, inform functional gain or loss. Using the PDX cohort and verifying trends in TCGA samples, we established a collection of GOE and LOE variants that strongly agree with GOF and LOF variants previously reported in the literature. Observed opposing expression patterns provide a framework to prioritize variants for future experimental followup in PDX models or other pre-clinical systems. Analysis of RPPA data in isolation did not identify significant associations, albeit top ranked proteins were enriched for melanoma-relevant pathways (Extended Data Figure S7). Thus, integration of RPPA with other data types throughout this study allowed derivation of meaningful associations (Figures 3D–3F and 4). We also identified a link between somatic GOF/GOE and germline LOF/LOE variants, with potential for tumor-specific targeting of GOF/GOE mutant. This pattern is observed at the pathway level, revealing additional opportunities to target tumor-gained signaling processes. Negative regulation of GOF/GOE pathways was associated with treatment response, offering a route to explore predictive regulatory patterns.

We further characterized GOF and LOF melanoma variants using multi-omics factor analysis, integrating variant, gene, and protein contributions within latent factors. While expression signatures can suggest functional effects, not all GOF or LOF variants alter transcription. Some impact protein stability, interactions, or pathway activity. This analysis captures observed or latent coregulation patterns rather than direct causal effects, which require experimental validation. Despite these limitations, our approach shows strong agreement with literature-reported GOF/LOF variants and can distinguish GOE from LOE and GOF from LOF. Incorporating structural context further refined these predictions and enabled identification of new candidate GOF variants in *KANSL1* and *HER3* that are not apparent from expression changes alone.

Interestingly, we found that larger structural contexts were more robust when predicting GOE/LOE variants, while smaller embedding dimensions performed well with less structural information when predicting GOF/LOF in literature-reported variants. This could imply that variants leading to either GOE or LOE in cancer are similar among many neighboring residues across a region of the protein. In this study, we employed PCA to the structural context representation for downstream prediction tasks, which may not be ideally suited for this purpose as it does not preserve spatial relationships. Given the consistent nonrandom performance we observe with this simple approach, we believe that optimizing dimensionality reduction in future studies could lead to highly accurate distinction of GOF from LOF variants in cancer.

In summary, this study provides melPDomiX, a unique multi-omics cohort of advanced melanoma PDX models that are available to the scientific community to support comprehensive pre-clinical melanoma research. We demonstrate the utility of this cohort by applying complementary approaches to classify melanoma variants into GOF and LOF categories. Our findings advance the understanding of the diverse genomic, molecular, and structural features associated with these functional variant classes, and the methodology described here may similarly facilitate characterization of variants in other cancer types. The GOF/GOE and LOF/LOE variants identified in this study have the potential to guide the development of new therapeutics targeting functional melanoma variants associated with resistance.

### Limitations of the study

Several limitations should be considered when interpreting findings from our study of the melPDomiX resource. Although melPDomiX is among the largest multi-omics melanoma PDX cohorts collected to date, the cohort is not adequately sized for stratified analyses involving treatment response and rarer, non-cutaneous subtypes, and associations identified in these subgroups will require validation in appropriately powered cohorts. Relationships between mutations and transcript or protein abundance are also correlative and may reflect coordinated genomic programs rather than direct causal effects of individual variants. The GOE and LOE framework captures variants with transcriptional or protein-level consequences but may not detect variants acting primarily through protein stability, post-translational modification, or protein-protein interactions. Structural-context analysis partially mitigates this limitation but is currently restricted to missense variants. In addition, integration of multi-omics data across platforms and batches may introduce residual technical variability despite normalization and correction procedures. Definitive functional classification for novel variants will thus require experimental validation in isogenic systems. Finally, as PDX models lack a human immune compartment, associations with immunotherapy response likely reflect tumor-intrinsic biology and should be confirmed in patient cohorts.

## RESOURCE AVAILABILITY

### Lead contact

Requests for further information and resources should be directed to and will be fulfilled by the lead contact, Noam Auslander (nauslander@wistar.org).

### Materials availability

All models are available from the lead contact upon request.

## STAR★METHODS

### EXPERIMENTAL MODEL AND STUDY PARTICIPANT DETAILS

Melanoma PDXs were established as previously described.^26,59,60^ In this study, we provide for the first time a multi-omics characterization of 242 of the PDXs.

## METHOD DETAILS

### Melanoma PDX generation and sample collection for melPDomiX

Tumor samples were collected under IRB approval and processed within 24 hours of biopsy. Samples were mechanically dissociated and, when necessary, enzymatically digested. Tumor tissue was either cryopreserved in 10% DMSO/90% FBS or implanted subcutaneously into anesthetized NSG mice with Matrigel through a small dorsal incision, which was subsequently closed with a wound clip. Tumor tissues from patients were aseptically dissected into fragments smaller than 1 mm^3^, which were then either implanted immediately into recipient NSG mice or cryopreserved in 10% DMSO/FBS in liquid nitrogen for future use. Under anesthesia, a ∼5 mm skin incision was made in the lower back of each mouse, and the tumor fragments, combined with 100 μL of Matrigel, were inserted into a subcutaneous pocket. The incision was then closed with wound clips. Tumor growth was monitored weekly using caliper measurements to calculate tumor volume (length × width^2^/2). Mice were euthanized when tumors reached approximately 1000 mm^3^ or earlier if necessary for animal welfare considerations. PDXs were harvested under sterile conditions. Harvested tumors were further dissected into small fragments. Most of these fragments were cryopreserved in 10% DMSO/FBS and stored in a live PDX tumor tissue biobank. The remaining fragments were snap-frozen in liquid nitrogen for subsequent DNA, RNA, and protein extraction. Formalin-fixed paraffin-embedded (FFPE) tumor blocks were also prepared. For PDX tumors derived from patients who had progressed on BRAFi or BRAFi/MEKi therapies, subsequent passages were maintained in mice on a continuous diet containing BRAFi or BRAFi/MEKi after one mouse passage. All patient samples were collected with informed consent and following Institutional Review Board (IRB) approvals. All animal experiments were conducted in compliance with protocols approved by the Institutional Animal Care and Use Committee (IACUC) of the Wistar Institute. All studies were performed in accordance with the Guide for Care and Use of Laboratory Animals published by the U.S. National Institute of Health under Institutional Animal Care and Use Committee protocol 201546. Wistar Animal Facility is fully accredited by the Association for Assessment and Accreditation of Laboratory Animal Care (AAALAC) and houses rodents for experimental research. This methodology ensures the rigorous and ethical generation of melanoma PDX models, providing a robust platform for ongoing cancer research and therapeutic development. Snap frozen PDX tumor tissue samples and FFPE blocks were sent to MD Anderson Cancer Center, Broad Institute and the Saint John Cancer Institute for protein, DNA and RNA analyses, respectively.

PDX models are available to the scientific community through Envigo Inc and directly from the Herlyn laboratory.

### melPDomiX Whole Exome sequencing (WES) dataset

#### WES sample collection.

WES was performed in the Broad Institute following its Twist Somatic Exome v6 methods. DNA was isolated from Snap frozen PDX tumor tissue. For library construction, an aliquot of genomic DNA (100-250ng in 50μL) was used as the input for DNA fragmentation (aka shearing). Shearing was performed acoustically using a Covaris focused-ultrasonicator, targeting 150bp fragments. Library preparation was performed using a commercially available kit provided by KAPA Biosystems (KAPA HyperPrep Kit with Library Amplification product KK8504) and IDT’s duplex UMI adapters. Unique 8-base dual index sequences embedded within the p5 and p7 primers (purchased from IDT) were added during PCR. Enzymatic clean-ups were performed using Beckman Coultier AMPure XP beads with elution volumes reduced to 30μL to maximize library concentration. Following library construction, library quantification was performed using the Invitrogen Quant-It broad range dsDNA quantification assay kit (Thermo Scientific Catalog: Q33130) with a 1:200 PicoGreen dilution. Following quantification, each library was normalized to a concentration of 35 ng/μL, using Tris-HCl, 10mM, pH 8.0. All steps performed during the library construction process and library quantification process were performed on the Agilent Bravo liquid handling system. For in-solution hybrid selection, after library construction, hybridization and capture were performed using the relevant components of IDT’s XGen hybridization and wash kit and following the suggested protocol of the manufacturer, with several exceptions. A set of 12-plex pre-hybridization pools were created. These pre-hybridization pools were created by equivolume pooling of the normalized libraries, Human Cot-1 and IDT XGen blocking oligos. The pre-hybridization pools underwent lyophilization using the Biotage SPE-DRY. Post lyophilization, custom exome bait (TWIST Biosciences) along with hybridization mastermix was added to the lyophilized pool prior to resuspension. Samples were incubated overnight. Library normalization and hybridization setup were performed on a Hamilton Starlet liquid handling platform, while target capture was performed on the Agilent Bravo automated platform. Post capture, a PCR was performed to amplify the capture material. For preparation of libraries for cluster amplification and sequencing, after post-capture enrichment, library pools were quantified using qPCR (automated assay on the Agilent Bravo), using a kit purchased from KAPA Biosystems with probes specific to the ends of the adapters. Based on qPCR quantification, pools were normalized using a Hamilton Starlet to the required loading concentration. Up to 24 samples were sequenced per lane on Illumina NovaSeq S4 sequencing technology. For cluster amplification and sequencing, cluster amplification of library pools was performed according to the protocol of the manufacturer (Illumina) using Exclusion Amplification cluster chemistry and NovaSeq S4 flowcells. Flowcells were sequenced on Sequencing-by-Synthesis chemistry for NovaSeq S4 flowcells using paired 151bp runs.

### melPDomiX RNA sequencing (RNA-seq) dataset

#### RNA-seq sample collection.

PDX snap frozen samples were performed at the Broad Institute following Broad Stranded RNA-Seq Method. For cDNA Library Construction, total RNA was quantified using the Quant-iT^™^ RiboGreen^®^ RNA Assay Kit and normalized to 5ng/uL. Following plating, 2 uL of ERCC controls (using a 1:1000 dilution) were spiked into each sample. An aliquot of 325 ng for each sample was transferred into library preparation which uses automated variant of the Illumina TruSeq^™^ Stranded mRNA Sample Preparation Kit. This method preserves strand orientation of the RNA transcript. It uses oligo dT beads to select mRNA from the total RNA sample. It is followed by heat fragmentation and cDNA synthesis from the RNA template. The resultant 400bp cDNA then goes through dual-indexed library preparation: ‘A’ base addition, adapter ligation using P7 adapters, and PCR enrichment using P5 adapters. After enrichment the libraries were quantified using Quant-iT PicoGreen (1:200 dilution). After normalizing samples to 5 ng/uL, the set was pooled and quantified using the KAPA Library Quantification Kit for Illumina Sequencing Platforms. The entire process is in 96-well format and all pipetting is done by either Agilent Bravo or Hamilton Starlet. For Illumina Sequencing, pooled libraries were normalized to 2nM and denatured using 0.1 N NaOH prior to sequencing. Flowcell cluster amplification and sequencing were performed according to the protocols of the manufacturer using NovaSeq. Each run was a 151bp paired-end with an eight-base index barcode read. Data was analyzed using the Broad Picard Pipeline which includes de-multiplexing and data aggregation. An aliquot of 325 ng for each sample was transferred into library preparation using automated variant of the Illumina TruSeq^™^ Stranded mRNA Sample Preparation Kit. It was followed by heat fragmentation and cDNA synthesis from the RNA template. The resultant 400bp cDNA then went through dual-indexed library preparation: ‘A’ base addition, adapter ligation using P7 adapters, and PCR enrichment using P5 adapters. After enrichment, the libraries were quantified using Quant-iT PicoGreen (1:200 dilution). After normalizing samples to 5 ng/uL, the set was pooled and quantified using the KAPA Library Quantification Kit for Illumina Sequencing. Pooled libraries were normalized to 2nM and denatured using 0.1 N NaOH prior to sequencing. Flowcell cluster amplification and sequencing were performed according to the protocols of the manufacturer through NovaSeq. Each run was a 101bp paired-end with an eight-base index barcode read. Data was analyzed using the Broad Picard Pipeline which includes de-multiplexing and data aggregation.

### melPDomiX reverse protein phase assay (RPPA) dataset

#### RPPA sample collection.

Snap frozen PDX tumor tissue was used to performed RPPA in the RPPA Core Facility, MD Anderson Cancer Center, as described previously.^49^ Cellular proteins were denatured in a 1% SDS + 2-mercaptoethanol buffer solution and diluted in five 2- fold serial dilutions in dilution lysis buffer. Serially diluted lysates were arrayed on nitrocellulose-coated slides (Grace Bio-Labs) by the Quanterix (Aushon) 2470 Arrayer (Quanterix Corporation). A total of 5808 spots were arrayed on each slide including spots corresponding to serially diluted (1) standard lysates, and (2) positive and negative controls prepared from mixed cell lysates or dilution buffer, respectively. Each slide was probed with a validated primary antibody plus a biotin-conjugated secondary antibody. Antibody validation for RPPA is described in the RPPA Core website: https://www.mdanderson.org/research/research-resources/core-facilities/functional-proteomics-rppa-core.html. Signal detection was amplified using an Agilent GenPoint staining platform (Agilent Technologies) and visualized by DAB colorimetric reaction. The slides were scanned (Huron TissueScope, Huron Digital Pathology) and quantified using customized software (Array-Pro Analyzer, Media Cybernetics) to generate spot intensity. Relative protein level for each sample was determined by RPPA SPACE (developed by MD Anderson Department of Bioinformatics and Computational Biology^61^) by which each dilution curve was fitted with a logistic model. RPPA SPACE fits a single curve using all the samples (i.e., dilution series) on a slide with the signal intensity as the response variable and the dilution steps as the independent variable. The fitted curve is plotted with the signal intensities, both observed and fitted, on the y-axis and the log2 concentration of proteins on the x-axis for diagnostic purposes. The protein concentrations of each set of slides were then normalized for protein loading. Correction factor was calculated by (1) median-centering across samples of all antibody experiments; and (2) median-centering across antibodies for each sample. Results were then normalized across RPPA sets by replicates-based normalization as described previously.^62^ Of note, we removed primary antibodies of mouse origin from the cohort, and all antibodies have been validated to bind to the human antigen prior to inclusion in the assays.

### *In vivo* validation of melanoma response to BRAF/MEK inhibition

Human melanoma cells derived from three patient-derived xenografts (PDXs), two harboring the *ANAPC4* R465Q mutation (WM4007 and WM4237) and one *ANAPC4* wild-type tumor (WM425), were injected subcutaneously into NSG mice. Tumor growth was monitored by caliper measurements of tumor length and width, and tumor volume was calculated accordingly. Once tumors reached approximately 200 mm^3^, mice were randomized to receive either Dabrafenib/Trametinib-formulated chow or a control diet. Tumor volumes were measured longitudinally to generate growth curves, which were used to compare the responsiveness of different melanoma models to BRAF/MEK inhibition (Extended Data Figure S1D).

## QUANTIFICATION AND STATISTICAL ANALYSIS

### melPDomiX data processing

PDX samples were sequenced in five batches and resulting fastq processed read files were obtained after quality control. Processed reads for PDX samples were aligned to the *mus musculus* (mm10) genome with bowtie2^51^ (version 2.2.9) to be excluded. Unaligned reads were then aligned to the GRCh38 human genome using Burrows-Wheeler Aligner^63^ (BWA-mem version 0.7.17-r1188) to generate .bam files to initiate variant calling. For PDXs without available matched normal blood samples, using picard (version 2.26.10), sambamba^56^ (version 0.8.1), samtools^63^ (version 1.15.1) with bcftools (version 1.14) for variant calling, reads were first sorted, and mate-pair information was fixed. For PDXs with available matched normal blood samples, similar pre-processing steps to generate relevant .vcf files were conducted, where variants were instead called using VarScan2,^57^ with tumor samples and corresponding normal samples processed as pairs. All resulting variants in both PDXs with and without matched pairs were annotated with SnpEff^64^ 4.3t (build 2017-11-24 10:18), using GRCh38.86 as reference. Additional filtering parameters set for WES processing are described in the Supplementary material.

### RNA-seq data processing

PDX samples were sequenced in 5 batches, and adapter sequences in .fastq raw read files were trimmed using jgi.BBDuk from BBTools^65^ (Version 38.44). Reads in trimmed .fastq files were then aligned to the mm10 transcriptome using bowtie2^66^ (version 1.3.1). Similar to WES processing, trimmed reads for PDX samples that aligned to the mm10 transcriptome with bowtie2 (version 1.3.1) were removed. Subsequent reads that aligned to the human transcriptome using bowtie2^51^ (version 2.2.9) were quantified using RNA-seq by Expectation Maximization (RSEM) (version 1.3.1) to generate count files. ENSEMBL ID gene names in RSEM data were converted to original gene symbols, and sample indices were harmonized to align with other omics datasets. Using fragments per kilobase of transcript per million mapped reads (FPKM) normalization, we obtained normalized read counts for 56,518 genes across batches. Genes with sparse expression and near-zero variance were excluded.

### RPPA data processing

PDX samples were assayed across 6 batches through the Functional Proteomics RPPA Core supported by MD Anderson Cancer Center. All resulting data were normalized for protein loading and transformed to linear and log2 median centered values. Negative controls, tissue reactive antibodies, and all antibodies produced in mouse and rat were removed. Validated antibodies and antibodies that had good staining (defined as having quality control metric greater than or equal to 0.8) were included in the dataset. Since RPPA data was merged across 6 batches, protein loading correction and antibody variation adjustment was assessed by excluding samples deemed protein concentration outliers (defined as a sample having less than 0.25 or greater than 2.5 protein loading correction factor in the entire RPPA dataset). Quality control and filtering resulted in 462 total antibodies.

### melPDomiX evaluation of copy number alteration across genes

Aligned bam files from PDXs that were generated for mutation calling were used as input to establish copy number alterations. The CNVkit^53^ Python package was utilized to call CNA without matched normal from aligned bam files across PDXs (Table S10). In agreement with previous studies,^18,67^ we found a lower frequency of copy number alteration events in melanoma (Table S10), in contrast to a very high point mutation rate.^27^ These findings provide further justification to perform variant expression-based analysis, which showed strong agreement with previously reported GOF and LOF variants.

### Data normalization and batch correction

RNA-seq and RPPA from PDXs were quantile normalized to adjust sample-wise distributions to be the same, assuming global differences in the distribution were induced by technical variation.^68,69^ Batch effect adjusted RNA-seq data were generated by estimating batch free distributions using ComBat-seq negative binomial regression model parameter estimates.^54^ This was computed using the pyCombat package^55^ in Python version 3.9.12 on log_2_(x+1) transformed data. Batch effects in RPPA data were regressed away using a limma gene-wise linear model, conducted in R version 4.3.2 using the removeBatchEffects function, also on log_2_(x+1) transformed data.^16^ Combinations of each correction method were employed to acquire the most well-mixed batches across PDX clusters. Principal component analysis (PCA) was conducted to visually assess batch effects in single-omics modalities post normalization.

### TCGA SKCM data

Somatic mutation data and RNA sequencing data were obtained from TCGA SKCM^18^ through Xena Browser,^50^ using VarScan2 Variant calling for somatic mutations, and HTSeq FPKM processed mRNA. Germline mutations from TCGA were extracted through a previous study,^70^ where access to VCF file was obtained from the Genome Data Commons (GDC) after receiving permission via dbGaP.

### Annotating GOE and LOE variants by gene expression fold changes

To classify GOE and LOE variants using directions of increased or decreased expression, average log2 fold changes were calculated between mutant and wildtype PDX subgroups for the corresponding gene or protein of the variant. Due to most tumor types being cutaneous melanomas, all subtypes were pooled together for calculation of effect sizes and assumed to be independent. The change in expression direction was used because most variants are too rare to conduct adequately powered hypothesis testing under multiple comparisons given the observed effect size, including known GOF melanoma variants (Figure 1D, Extended Data Figure S8). Therefore, while the increase or decrease in expression might show consistent GOE/LOE associations, respectively, use of differential expression testing for capturing this trend is likely underpowered and would lead to multiple false negatives. We therefore described observed effect sizes and found clear trends between variant presence and gene/protein expression patterns, with known driver mutations aligning well with expression-based classifications. Variants with positive and negative fold change in the PDX cohort which exhibited the same directionality in the TCGA cohorts were annotated as likely GOE and LOE variants, respectively.

### Pan cancer expression change analysis

Pan-cancer TCGA somatic mutation and gene expression data were obtained from the Xena Browser.^50^ Pan-cancer driver gene annotations were obtained from Bailey et al.^31^ which classify genes as tumor suppressors, characterized by loss-of-function mutations, or oncogenes, characterized by gain-of-function mutations. For each gene, we examined the fold change in expression between samples in which the gene was mutated and samples in which the gene was not mutated, considering missense mutations only. Fold-change values were calculated and visualized for each gene across cancer types (Extended Data Figure S2A).

### Variants annotation as somatic or germline

Variants were annotated as likely somatic or germline using both the melPDomiX and TCGA cohort. Through the melPDomiX cohort, we compared for each variant (1) the frequency it was called only without matched blood and not using matched blood samples through VARSCAN2 to (2) the frequency it was called both with and without matched blood, in the same PDX. Variants that were more frequently called only without matched blood were annotated as germline, whereas variants that were more frequently called with matched blood through VARSCAN2 were annotated as somatic.

For TCGA data, we used the germline and somatic called mutations to evaluate variant frequency of occurring as germline vs. somatic. We observe 100% agreement between germline vs. somatic variant classification for the variants that we were able to annotate as such using both datasets (Table S8).

### Cross reference of variants with SNP databases

Reference SNP cluster IDs (RSIDs) from human genome build 38 variant positions for variants in *CTNNB1*, *ATM*, *TM9SF1*, *HLA-DPB1*, *ANAPC4*, and *MSH3* were obtained from dbSNP. Allele frequencies from non-Finnish European and non-cancer non-Finnish European populations were reported from gnomAD v2.1.1 (GRCh 38 lifted) exomes. Traits reported in the NHGRI-EBI GWAS Catalog^71^ as of November 2025 were denoted for all variants. There were 125 total associations for the *ANAPC4* rs34811474 variant spanning a wide range of phenotype categories, including measurements related to vitals, laboratory tests, and sociodemographic factors. For readability, we report only the most relevant and most frequently associated traits for this variant. The tissue with the highest median bulk expression for each gene containing the variant of interest was obtained from GTEx Analysis Release V10 (dbGaP Accession phs000424.v10.p2). We additionally cross-referenced the traits of interest in ClinVar,^72^ OncoKB,^73^ and MelanoDB,^74^ a database of clinical and molecular features of patients with melanoma treated with MAPK inhibitors. All three databases were accessed in November 2025 (Table S12).

### Pathway analysis

Pathway enrichment was applied to GOE, LOE, germline and somatic annotated variants by employing a Fisher’s exact test to evaluate enrichment between genes belonging to GOE, LOE, germline, or somatic associated variants and genes associated with every pathway (through five pathway annotations, Table S11). Pathway enrichment was additionally performed by calculating a pathway score using whole-exome sequencing (WES), RNA-sequencing (RNA-seq), and reverse-phase protein array (RPPA) data. For WES, mutations were aggregated by gene, and samples were considered to have a mutation in a gene if one or more mutated variants of the gene were present. The percentage of mutated genes in each pathway were then z-scored. For RNA-seq and RPPA, data were first z-scored by gene, and a pathway score for each sample was calculated as the mean z-score of the genes present in the pathway. Correlation matrices were calculated between pathways of each omics type for responders, non-responders, and those with mixed response/stable disease categories, using Spearman correlation using the scipy package in Python. This was done to allow direct comparison of regulation (by correlation strengths) within and between pathways across clinical response groups. Correlation plots were made using the corrplot package in R.

### Unsupervised multi-omics integration

WES, RNA-seq, and RPPA data were integrated using multi-omics factor analysis (MOFA^41^), to infer the latent representation of the three modalities and identify latent sources of biological variation between them. For sample i, modality h, covariate j per modality, and latent factor I, the following MOFA model was used (using the Gaussian data case as an example):

$$Y_{i}^{h}=W^{h}Z_{i}^{h}+\varepsilon_{i}^{h},$$

where Z∼N(0,l) is a L×n latent factor matrix with $L\leq min(n,p),\varepsilon ∼N\left(0,\tau^{-1}\right)$ is a p×n matrix, allowing heteroscedasticity across features, and $W={\left[w_{1}\ldots w_{p}\right]}^{T}$, where $w_{j}$ is the vector of L-dimensional factor loadings which weigh the contributions of the individual latent factors to the $j^{\text{th}}$ feature. A sparse prior imposed on W to induce shrinkage of the factor loadings. Variation explained $\left(R^{2}\right)$ of each modality was computed using the RV-coefficient as previously presented:

$$R_{lh}^{2}=1-{\left(\sum_{j,i}\;\;y_{ji}^{h}-w_{jl}z_{li}-\mu_{i}^{h}\right)}^{2}/{\left(\sum_{j,i}\;\;y_{ji}^{h}-\mu_{i}^{h}\right)}^{2},$$

where μ is related to the expectation of each omics layer $Y^{h}.\mu$ is modeled using a logit link for the WES data. MOFA was applied to both melPDomiX and TCGA SKCM data.

### GOF/GOE and LOF/LOE variant annotation through MOFA

Variants were additionally annotated as likely GOF/GOE or LOF/LOE based on the concordance of their latent factor loadings across omics (Extended Data Figure S3A). Candidate GOF/GOE or LOF/LOE variants were initially screened per factor by choosing variants with gene and protein products that had uniformly same or opposite loading sign. Since the factor loadings were sparse by design, variants with zero loading after within-factor standardization were excluded, as they do not contribute to the variability explained by each latent factor. For each variant per factor, the sum of the pairwise products of factor loadings for the variant and its corresponding gene and protein product acted as a measure of concordance across omics:

$$w_{j}^{\text{variant}\;}=w_{j}^{\text{WES}\;}w_{j}^{\text{RNA}\;}+w_{j}^{\text{WES}\;}w_{j}^{\text{RPPA}\;}+w_{j}^{\text{RNA}\;}w_{j}^{\text{RPPA}\;}.$$

Highly weighted variants with positive score had strongly positive correlation with the factor the variant loaded onto, denoting GOF/GOE status. Similarly, candidate LOF/LOE variants exhibited low score and strongly negative correlation with their respective factors across omics. The maximum absolute weighted score across factors was chosen as the representative score, i.e., such that across all L factors, $max\left(\left|w_{j}^{\text{variant}\;}\right|\right)\gg 0$ is assigned GOF/GOE, and $max\left(\left|w_{j}^{\text{variant}\;}\right|\right)\ll 0$ is assigned LOF/LOE. The final scores, $w^{\text{variant}\;}$, were used for downstream associations of likely GOE and LOE variants with pathway annotations.

### Variant representation using AlphaMissense

For each variant, the consensus and computationally mapped isoform reference sequences were extracted from the Uniprot database. AlphaMissense was then run on variants with single amino acid substitutions to extract the pair representation using default configurations (parameter *zero_init* was set to *False*). Residues of unknown amino acids (other than the twenty which comprise proteins) were defined as ‘X’ for one-hot encoding.

Each sequence was preprocessed in the data pipeline of AlphaMissense to determine whether spatial or contiguous region cropping could be used. If the reference sequence had known predicted protein structures in the AlphaFold Protein Structure Database, the atomic coordinates for the AlphaFold predicted reference sequence structure would be used for spatial cropping.^75,76^ Spatial regions used the 256 closest residues to the variant residue by atomic distance. If the predicted structure could not be determined, the contiguous region of 256 amino acids centered around the variant position was used instead. This process was also repeated to embed the sequences using the 8 closest residues to the variant residue by spatial coordinates to investigate whether reducing dimensionality would help differentiate between literature-reported GOF/LOF labels. The MSA search used the default dependencies and the recommended sequence databases of AlphaMissense. The pair representation was extracted once the pipeline terminated, after 3 recycling iterations.

### GOE vs. LOE variant annotation through AlphaMissense representation

To use AlphaMissense pair representation for prediction of gain- and loss-of-function variants, a training dataset with 14 loss and 19 gain-of-function variants was utilized (Table S9). Each sample was flattened per row, scaled according to the IQR and median, to account for extreme outlier logits, and then dimensionally reduced using PCA (using the scikit-learn package in Python). The first four principal components were then extracted and used to train K-Nearest Neighbors classifiers (with k=3), across 500 randomized splits. In every split, 75% of the samples were used for training and 25% as validation (for performance evaluation). An average score per training variant was calculated by counting the total correctly predicted labels across splits.

### Prediction of new GOF/LOF variants

To perform supervised prediction of potentially novel gain- and loss-of-function variants, a candidate set (n=32) of 17 GOE variants and 15 LOE variants was extracted through the top predicted MOFA annotations. The scaler and PCA which were fitted using the training set, were used to transform the test set variants. A K-Nearest Neighbors Classifier and BaggingRegressor were trained to cross reference the labels and weights obtained through the expression-based GOF and LOF annotations from MOFA (Extended Data Figure S4).

In addition, a supervised prediction of literature-reported GOF/LOF variants (n=11) using 8x8x4 AlphaMissense embeddings was employed to evaluate performance on literature-reported variants classified previously as GOF/LOF. A RandomizedGridSearch (using scikit-learn package in Python) was used to hypertune parameters for a RandomForestClassifier and calculate the ROC AUC across 500 random seeds to evaluate performance (Extended Data Figure S5B).

### PDX Whole Exome sequencing (WES) dataset - Mutation calling

Reads were mapped using parameters -q 30 -F 0x800 -F 0x100 -F 4 and mapping_quality ≥ 1 and not (unmapped or secondary_alignment) and not ([XA] != null or [SA] != null while removing read duplicates. Final filtering to call variants and generate .vcf files included the following specification: -s LowQual -g3 -G10 -e’%QUAL<10 ‖ (RPBZ<0.1 && %QUAL<15) ‖ (AC<2 && %QUAL<15) ‖ MQ < 30 ‖ MQSBZ ≤0.1 ‖ %MAX(DV)<=3 ‖ %MAX(DV)/%MAX(DP)<=0.3.

Variants in .vcf files were then overlapped with both COSMIC somatically acquired mutations and mutations called in the 1000 Genomes Project database. The final processed variants consisted of those that overlapped with COSMIC mutations but did not overlap with 1000 Genomes Project, or variants that were not found in either database. Variants that were found within the 1000 Genomes Project reference were thus fully excluded regardless of allele frequency to remove common variants shared in healthy individuals.

The final variant list was categorized by the following point mutation types: synonymous variant, missense variant, 3-prime UTR variant, 5-prime UTR variant, 5-prime UTR premature start codon gain variant, stop gained, stop gained and splice region variant, stop lost, start lost, frameshift variant, disruptive in-frame deletion, and disruptive in-frame insertion.

409,933 variants were called in 19,877 genes across both types of PDX. Variants with less than or equal to 1% frequency among samples were removed. Due to a majority of the PDX lines not having matched normal blood samples available, mutations were not able to be distinguished as either somatic or germline mutations for all samples. Although germline variants specifically can exhibit causal relationships with melanomagenesis, mutations in oncogenes that are either somatically acquired or inherited can drive cancer and lead to increased cancer cell proliferation. We thus focused on a subset of frequent variants in known driver genes, regardless to whether they are more likely germline or somatic. This subset included *BRAF* pV600x variants, *ARID1A* deletion or nonsense mutation, and more, reducing the original dimension to 240 variants in 132 driver genes. The final WES dataset was converted into a binary matrix with 3-prime UTR variant, 5-prime UTR variant, and synonymous variants labeled as 0 and all other mutation types (possibly deleterious) labeled as 1 for each sample.

### Linking gene variants with treatment responses

Gene variants were linked with treatment benefit using a hypergeometric enrichment test. The enrichment between each variant and PDX from patients who benefitted from immunotherapy or targeted therapy was tested. PDX from patients showing complete or partial responses, mixed responses and stable disease were grouped as treatment benefit, whereas patients with progressive disease were deemed non-treatment benefit. Hypergeometric enrichment p-values were calculated for variants occurring in at least 30% and at most 70% of the PDX with patient treatment response information. P-values were then FDR-corrected for multiple comparisons, and corrected q<0.1 were further considered. The only corrected significant BRAFi/MEKi benefit association identified was *ANAPC4* R465Q, and the top significant non-BRAFi/MEKi benefit associations were *HLA-DPB1* variants (Extended Data Figure S1). No significant association with immunotherapy benefit was identified.

### Prior associations of treatment response linked variants

We performed an association analysis of these SNPs. Independent validation of their relationship with clinical response to BRAF inhibition is limited by the absence of publicly available cohorts that include both somatic variant profiles and detailed treatment response data. While rs34811474 in *ANAPC4* has no reported cancer- or immune-related associations, it has been linked to osteoarthritis,^30^ where BRAF inhibition was recently shown to alleviate cartilage degradation.^29^ Other *ANAPC4* variants have been linked to RAF inhibition in melanoma.^77^ For *HLA-DPB1*, the G40V variant (rs1126513) may influence mTORC1 signaling, a pathway modulated by BRAF/MEK inhibitors, providing a biologically plausible connection. These observations highlight candidate variants that merit further investigation, even in the absence of large-scale replication datasets, and suggest that these likely common haplotypes could represent potential inherited resistance mechanisms to BRAFi/MEKi therapy (Extended Data Figure S1A; Tables S2 and S3, Fisher’s exact test FDR q<0.1).

## Supplementary Material

1

2

3

4

5

6

7

8

9

10

11

12

13

Supplemental information can be found online at https://doi.org/10.1016/j.celrep.2026.117455.

## Figures and Tables

**Table:** KEY RESOURCES TABLE

REAGENT or RESOURCE	SOURCE	IDENTIFIER
Antibodies		
RPPA pipeline	Siwak et al.^49^	https://www.mdanderson.org/research/research-resources/core-facilities/functional-proteomics-rppa-core.html
Deposited data		
Somatic mutation data and RNA sequencing data	Goldman et al.^50^	https://xenabrowser.net/datapages/?cohort=GDC%20TCGA%20Melanoma%20(SKCM)&removeHub=https%3A%2F%2Fxena.treehouse.gi.ucsc.edu%3A443
Pan-cancer TCGA somatic mutation and gene expression data	Goldman et al.^50^	https://xenabrowser.net/datapages/?cohort=TCGA%20Pan-Cancer%20(PANCAN)&removeHub=https%3A%2F%2Fxena.treehouse.gi.ucsc.edu%3A443
Pan-cancer driver gene annotations	Bailey et al.^31^	https://www.cell.com/cell/fulltext/S0092-8674(18)30237-X?_returnURL=https%3A%2F%2Flinkinghub.elsevier.com%2Fretrieve%2Fpii%2FS009286741830237X%3Fshowall%3Dtrue
Reference SNP cluster IDs	dbSNP	https://www.ncbi.nlm.nih.gov/snp/
Tissue-specific bulk RNA-seq expression data	dbGAP	phs000424.v10.p2
Consensus and computationally mapped isoform reference sequences	Uniprot	https://www.uniprot.org/
AlphaFold Protein Structure Database	Google DeepMind; EMBL-EBI	https://alphafold.ebi.ac.uk/
RNA-sequencing data	This study	GEO: GSE297199
Whole-exome sequencing data	This study	GEO: GSE297200
Reverse phase protein assay data	This study	GEO: GSE298502
Experimental models: Organisms/strains		
NSG mice	Krepler et al.^26^	https://pmc.ncbi.nlm.nih.gov/articles/PMC5726788/
Software and algorithms		
jgi.BBDuk	BBTools	https://archive.jgi.doe.gov/data-and-tools/software-tools/bbtools/bb-tools-user-guide/bbduk-guide/
Bowtie (1.3.1)	Langmead & Salzberg.^51^	https://bowtie-bio.sourceforge.net/index.shtml
RNA-seq by Expectation Maximization (RSEM, 1.3.1)	Li & Dewey^52^	https://github.com/deweylab/RSEM
CNVkit	Talevich et al.^53^	https://cnvkit.readthedocs.io/en/stable/
ComBat-seq	Zhang et al.^54^	https://github.com/zhangyuqing/ComBat-seq
pyCombat	Behdenna et al.^55^	https://github.com/epigenelabs/pyComBat
Multi-omics factor analysis (MOFA)	Argelaguet et al.^41^	https://biofam.github.io/MOFA2/index.html
AlphaMissense	Cheng et al.^43^	https://alphamissense.hegelab.org/
picard	Broad Institute	https://broadinstitute.github.io/picard/
sambamba	Tarasov et al.^56^	https://github.com/biod/sambamba
VaraScan 2	Koboldt et al.^57^	https://varscan.sourceforge.net/
COSMIC database	Sondka et al.^58^	https://pmc.ncbi.nlm.nih.gov/articles/PMC10767972/

## Data Availability

The melPDomiX multi-omics cohort and respective clinical information are available through the supplementary information, via Tables S1, S2, S3, S5, and S6. The three omics dataset have been additionally deposited into GEO with accessions GSE297199, GSE297200, and GSE298502, all of which will be made public upon acceptance of this work. Code implemented for this study is available through GitHub: https://github.com/AuslanderLab/melPDomiX. Code is also available through Zenodo: https://doi.org/10.5281/zenodo.19485537.

## Highlights

- melPDomiX provides multi-omics melanoma PDX models with available tumor resources
- Integrated genomics, transcriptomics, and proteomics map the functional impact of mutations
- Structural-context analysis distinguishes gain- from loss-of-function variants

## References

[1] Hodis E, Watson IR, Kryukov GV, Arold ST, Imielinski M, Theurillat JP, Nickerson E, Auclair D, Li L, Place C, (2012). A landscape of driver mutations in melanoma. Cell 150, 251–263. 10.1016/j.cell.2012.06.024. 22817889 PMC3600117

[2] Lawrence MS, Stojanov P, Polak P, Kryukov GV, Cibulskis K, Sivachenko A, Carter SL, Stewart C, Mermel CH, Roberts SA, (2013). Mutational heterogeneity in cancer and the search for new cancer-associated genes. Nature 499, 214–218. 10.1038/nature12213. 23770567 PMC3919509

[3] Arnold M, Singh D, Laversanne M, Vignat J, Vaccarella S, Meheus F, Cust AE, de Vries E, Whiteman DC, and Bray F (2022). Global Burden of Cutaneous Melanoma in 2020 and Projections to 2040. JAMA Dermatol. 158, 495–503. 10.1001/jamadermatol.2022.0160. 35353115 PMC8968696

[4] Whiteman DC, Green AC, and Olsen CM (2016). The Growing Burden of Invasive Melanoma: Projections of Incidence Rates and Numbers of New Cases in Six Susceptible Populations through 2031. J. Invest. Dermatol 136, 1161–1171. 10.1016/j.jid.2016.01.035. 26902923

[5] Bastian BC (2014). The molecular pathology of melanoma: an integrated taxonomy of melanocytic neoplasia. Annu. Rev. Pathol 9, 239–271. 10.1146/annurev-pathol-012513-104658. 24460190 PMC4831647

[6] Volkovova K, Bilanicova D, Bartonova A, .Letaţsiová S, and Dusinska M (2012). Associations between environmental factors and incidence of cutaneous melanoma. Review. Environ. Health 11, S12. 10.1186/1476-069x-11-s1-s12. 22759494 PMC3388446

[7] Potrony M, Badenas C, Aguilera P, Puig-Butille JA, Carrera C, Malvehy J, and Puig S (2015). Update in genetic susceptibility in melanoma. Ann. Transl. Med 3, 210. 10.3978/j.issn.2305-5839.2015.08.11. 26488006 PMC4583600

[8] Bataille V (2003). Genetic epidemiology of melanoma. Eur. J. Cancer 39, 1341–1347. 10.1016/s0959-8049(03)00313-7. 12826035

[9] Bernard JJ, Gallo RL, and Krutmann J (2019). Photoimmunology: how ultraviolet radiation affects the immune system. Nat. Rev. Immunol 19, 688–701. 10.1038/s41577-019-0185-9. 31213673

[10] Hart PH, and Norval M (2018). Ultraviolet radiation-induced immunosuppression and its relevance for skin carcinogenesis. Photochem. Photobiol. Sci 17, 1872–1884. 10.1039/c7pp00312a. 29136080

[11] Grzywa TM, Paskal W, and Włodarski PK (2017). Intratumor and Intertumor Heterogeneity in Melanoma. Transl. Oncol 10, 956–975. 10.1016/j.tranon.2017.09.007. 29078205 PMC5671412

[12] Saginala K, Barsouk A, Aluru JS, Rawla P, and Barsouk A (2021). Epidemiology of Melanoma. Med. Sci 9, 63. 10.3390/medsci9040063. PMC8544364 34698235

[13] Switzer B, Puzanov I, Skitzki JJ, Hamad L, and Ernstoff MS (2022). Managing Metastatic Melanoma in 2022: A Clinical Review. JCO Oncol. Pract 18, 335–351. 10.1200/op.21.00686. 35133862 PMC9810138

[14] Tsai J, Lee JT, Wang W, Zhang J, Cho H, Mamo S, Bremer R, Gillette S, Kong J, Haass NK, (2008). Discovery of a selective inhibitor of oncogenic B-Raf kinase with potent antimelanoma activity. Proc. Natl. Acad. Sci. USA 105, 3041–3046. 10.1073/pnas.0711741105. 18287029 PMC2268581

[15] Chapman PB, Hauschild A, Robert C, Haanen JB, Ascierto P, Larkin J, Dummer R, Garbe C, Testori A, Maio M, (2011). Improved survival with vemurafenib in melanoma with BRAF V600E mutation. N. Engl. J. Med 364, 2507–2516. 10.1056/NEJMoa1103782. 21639808 PMC3549296

[16] Villanueva J, Vultur A, Lee JT, Somasundaram R, Fukunaga-Kalabis M, Cipolla AK, Wubbenhorst B, Xu X, Gimotty PA, Kee D, (2010). Acquired resistance to BRAF inhibitors mediated by a RAF kinase switch in melanoma can be overcome by cotargeting MEK and IGF-1R/PI3K. Cancer Cell 18, 683–695. 10.1016/j.ccr.2010.11.023. 21156289 PMC3026446

[17] Robert C, Karaszewska B, Schachter J, Rutkowski P, Mackiewicz A, Stroiakovski D, Lichinitser M, Dummer R, Grange F, Mortier L, (2015). Improved overall survival in melanoma with combined dabrafenib and trametinib. N. Engl. J. Med 372, 30–39. 10.1056/NEJMoa1412690. 25399551

[18] Cancer Genome Atlas Network (2015). Genomic Classification of Cutaneous Melanoma. Cell 161, 1681–1696. 10.1016/j.cell.2015.05.044. 26091043 PMC4580370

[19] Chambers CA, Sullivan TJ, and Allison JP (1997). Lymphoproliferation in CTLA-4-deficient mice is mediated by costimulation-dependent activation of CD4+ T cells. Immunity 7, 885–895. 10.1016/s1074-7613(00)80406-9. 9430233

[20] Ishida Y, Agata Y, Shibahara K, and Honjo T (1992). Induced expression of PD-1, a novel member of the immunoglobulin gene superfamily, upon programmed cell death. Embo j 11, 3887–3895. 10.1002/j.1460-2075.1992.tb05481.x. 1396582 PMC556898

[21] Robert C, Thomas L, Bondarenko I, O’Day S, Weber J, Garbe C, Lebbe C, Baurain JF, Testori A, Grob JJ, (2011). Ipilimumab plus dacarbazine for previously untreated metastatic melanoma. N. Engl. J. Med 364, 2517–2526. 10.1056/NEJMoa1104621. 21639810

[22] Robert C, Ribas A, Wolchok JD, Hodi FS, Hamid O, Kefford R, Weber JS, Joshua AM, Hwu WJ, Gangadhar TC, (2014). Anti-programmed-death-receptor-1 treatment with pembrolizumab in ipilimumab-refractory advanced melanoma: a randomised dose-comparison cohort of a phase 1 trial. Lancet 384, 1109–1117. 10.1016/s0140-6736(14)60958-2. 25034862

[23] Lamba N, Ott PA, and Iorgulescu JB (2022). Use of First-Line Immune Checkpoint Inhibitors and Association With Overall Survival Among Patients With Metastatic Melanoma in the Anti-PD-1 Era. JAMA Netw. Open 5, e2225459. 10.1001/jamanetworkopen.2022.25459. 36006646 PMC9412220

[24] Wolchok JD, Chiarion-Sileni V, Gonzalez R, Grob JJ, Rutkowski P, Lao CD, Cowey CL, Schadendorf D, Wagstaff J, Dummer R, (2022). Long-Term Outcomes With Nivolumab Plus Ipilimumab or Nivolumab Alone Versus Ipilimumab in Patients With Advanced Melanoma. J. Clin. Oncol 40, 127–137. 10.1200/jco.21.02229. 34818112 PMC8718224

[25] Robert C, Grob JJ, Stroyakovskiy D, Karaszewska B, Hauschild A, Levchenko E, Chiarion Sileni V, Schachter J, Garbe C, Bondarenko I, (2019). Five-Year Outcomes with Dabrafenib plus Trametinib in Metastatic Melanoma. N. Engl. J. Med 381, 626–636. 10.1056/NEJMoa1904059. 31166680

[26] Krepler C, Sproesser K, Brafford P, Beqiri M, Garman B, Xiao M, Shannan B, Watters A, Perego M, Zhang G, (2017). A Comprehensive Patient-Derived Xenograft Collection Representing the Heterogeneity of Melanoma. Cell Rep. 21, 1953–1967. 10.1016/j.celrep.2017.10.021. 29141225 PMC5726788

[27] Alexandrov LB, Nik-Zainal S, Wedge DC, Aparicio SAJR, Behjati S, Biankin AV, Bignell GR, Bolli N, Borg A, Børresen-Dale AL, (2013). Signatures of mutational processes in human cancer. Nature 500, 415–421. 10.1038/nature12477. 23945592 PMC3776390

[28] Chang MT, Asthana S, Gao SP, Lee BH, Chapman JS, Kandoth C, Gao J, Socci ND, Solit DB, Olshen AB, (2016). Identifying recurrent mutations in cancer reveals widespread lineage diversity and mutational specificity. Nat. Biotechnol 34, 155–163. 10.1038/nbt.3391. 26619011 PMC4744099

[29] Dong Y, Wang P, Zhang M, Xiao L, Yang Y, Wang B, Liu Y, Dai Z, and Zheng J (2022). Phosphoproteomics reveals the BRAF-ERK1/2 axis as an important pathogenic signaling node in cartilage degeneration. Osteoarthr. Cartil 30, 1443–1454. 10.1016/j.joca.2022.08.003. 36100125

[30] Aubourg G, Rice SJ, Bruce-Wootton P, and Loughlin J (2022). Genetics of osteoarthritis. Osteoarthr. Cartil 30, 636–649. 10.1016/j.joca.2021.03.002. PMC9067452 33722698

[31] Bailey MH, Tokheim C, Porta-Pardo E, Sengupta S, Bertrand D, Weerasinghe A, Colaprico A, Wendl MC, Kim J, Reardon B, (2018). Comprehensive Characterization of Cancer Driver Genes and Mutations. Cell 173, 371–385.e18. 10.1016/j.cell.2018.02.060. 29625053 PMC6029450

[32] Talseth-Palmer BA, Bauer DC, Sjursen W, Evans TJ, McPhillips M, Proietto A, Otton G, Spigelman AD, and Scott RJ (2016). Targeted next-generation sequencing of 22 mismatch repair genes identifies Lynch syndrome families. Cancer Med. 5, 929–941. 10.1002/cam4.628. 26811195 PMC4864822

[33] Ma X, Zhang B, and Zheng W (2014). Genetic variants associated with colorectal cancer risk: comprehensive research synopsis, meta-analysis, and epidemiological evidence. Gut 63, 326–336. 10.1136/gutjnl-2012-304121. 23946381 PMC4020522

[34] Elisei R, Cosci B, Romei C, Bottici V, Sculli M, Lari R, Barale R, Pacini F, and Pinchera A (2004). RET exon 11 (G691S) polymorphism is significantly more frequent in sporadic medullary thyroid carcinoma than in the general population. J. Clin. Endocrinol. Metab 89, 3579– 3584. 10.1210/jc.2003-031898. 15240649

[35] Casula M, Paliogiannis P, Ayala F, De Giorgi V, Stanganelli I, Mandalà M, Colombino M, Manca A, Sini MC, Caracò C, (2019). Germline and somatic mutations in patients with multiple primary melanomas: a next generation sequencing study. BMC Cancer 19, 772. 10.1186/s12885-019-5984-7. 31382929 PMC6683413

[36] Barrett JH, Iles MM, Harland M, Taylor JC, Aitken JF, Andresen PA, Akslen LA, Armstrong BK, Avril MF, Azizi E, (2011). Genome-wide association study identifies three new melanoma susceptibility loci. Nat. Genet 43, 1108–1113. 10.1038/ng.959. 21983787 PMC3251256

[37] Li J, Shi D, Li S, Shi X, Liu Y, Zhang Y, Wang G, Zhang C, Xia T, Piao HL, and Liu HX (2024). KEAP1 promotes anti-tumor immunity by inhibiting PD-L1 expression in NSCLC. Cell Death Dis. 15, 175. 10.1038/s41419-024-06563-3. 38413563 PMC10899596

[38] Mahabeleshwar GH, and Byzova TV (2007). Angiogenesis in melanoma. Semin. Oncol. 34, 555–565. 10.1053/j.seminoncol.2007.09.009. 18083379 PMC2365306

[39] Inamdar GS, Madhunapantula SV, and Robertson GP (2010). Targeting the MAPK pathway in melanoma: why some approaches succeed and other fail. Biochem. Pharmacol 80, 624–637. 10.1016/j.bcp.2010.04.029. 20450891 PMC2897908

[40] Wellbrock C, and Arozarena I (2016). The Complexity of the ERK/MAP-Kinase Pathway and the Treatment of Melanoma Skin Cancer. Front. Cell Dev. Biol 4, 33. 10.3389/fcell.2016.00033. 27200346 PMC4846800

[41] Argelaguet R, Velten B, Arnol D, Dietrich S, Zenz T, Marioni JC, Buettner F, Huber W, and Stegle O (2018). Multi-Omics Factor Analysis-a framework for unsupervised integration of multi-omics data sets. Mol. Syst. Biol 14, e8124. 10.15252/msb.20178124. 29925568 PMC6010767

[42] Gerasimavicius L, Livesey BJ, and Marsh JA (2022). Loss-of-function, gain-of-function and dominant-negative mutations have profoundly different effects on protein structure. Nat. Commun 13, 3895. 10.1038/s41467-022-31686-6. 35794153 PMC9259657

[43] Cheng J, Novati G, Pan J, Bycroft C, Žemgulyte A, Applebaum T, Pritzel A, Wong LH, Zielinski M, Sargeant T, (2023). Accurate proteome-wide missense variant effect prediction with AlphaMissense. Science 381, eadg7492. 10.1126/science.adg7492. 37733863

[44] Koolen DA, Pfundt R, Linda K, Beunders G, Veenstra-Knol HE, Conta JH, Fortuna AM, Gillessen-Kaesbach G, Dugan S, Halbach S, (2016). The Koolen-de Vries syndrome: a phenotypic comparison of patients with a 17q21.31 microdeletion versus a KANSL1 sequence variant. Eur. J. Hum. Genet 24, 652–659. 10.1038/ejhg.2015.178. 26306646 PMC4930086

[45] Hanker AB, Brown BP, Meiler J, Marí A, Jayanthan HS, Ye D, Lin CC., Akamatsu H, Lee KM, Chatterjee S, (2021). Co-occurring gain-of-function mutations in HER2 and HER3 modulate HER2/HER3 activation, oncogenesis, and HER2 inhibitor sensitivity. Cancer Cell 39, 1099–1114.e8. 10.1016/j.ccell.2021.06.001. 34171264 PMC8355076

[46] Tiwary S, Preziosi M, Rothberg PG, Zeitouni N, Corson N, and Xu L (2014). ERBB3 is required for metastasis formation of melanoma cells. Oncogenesis 3, e110. 10.1038/oncsis.2014.23. 25000258 PMC4150209

[47] Coté D, Eustace A, Toomey S, Cremona M, Milewska M, Furney S, Carr A, Fay J, Kay E, Kennedy S, (2018). Germline single nucleotide polymorphisms in ERBB3 and BARD1 genes result in a worse relapse free survival response for HER2-positive breast cancer patients treated with adjuvant based docetaxel, carboplatin and trastuzumab (TCH). PLoS One 13, e0200996. 10.1371/journal.pone.0200996. 30071039 PMC6071997

[48] Shtivelman E, Davies MQA, Hwu P, Yang J, Lotem M, Oren M, Flaherty KT, and Fisher DE (2014). Pathways and therapeutic targets in melanoma. Oncotarget 5, 1701–1752. 10.18632/onco-target.1892. 24743024 PMC4039128

[49] Siwak DR, Li J, Akbani R, Liang H, and Lu Y (2019). Analytical Platforms 3: Processing Samples via the RPPA Pipeline to Generate Large-Scale Data for Clinical Studies. Adv. Exp. Med. Biol 1188, 113–147. 10.1007/978-981-32-9755-5_7. 31820386

[50] Goldman MJ, Craft B, Hastie M, Repečcka K, McDade F, Kamath A, Banerjee A, Luo Y, Rogers D, Brooks AN, (2020). Visualizing and interpreting cancer genomics data via the Xena platform. Nat. Biotechnol 38, 675–678. 10.1038/s41587-020-0546-8. 32444850 PMC7386072

[51] Langmead B, and Salzberg SL (2012). Fast gapped-read alignment with Bowtie 2. Nat. Methods 9, 357–359. 10.1038/nmeth.1923. 22388286 PMC3322381

[52] Li B, and Dewey CN (2011). RSEM: accurate transcript quantification from RNA-Seq data with or without a reference genome. BMC Bioinformatics 12, 323. 10.1186/1471-2105-12-323. 21816040 PMC3163565

[53] Talevich E, Shain AH, Botton T, and Bastian BC (2016). CNVkit: Genome-Wide Copy Number Detection and Visualization from Targeted DNA Sequencing. PLoS Comput. Biol 12, e1004873. 10.1371/journal.pcbi.1004873. 27100738 PMC4839673

[54] Zhang Y, Parmigiani G, and Johnson WE (2020). ComBat-seq: batch effect adjustment for RNA-seq count data. NAR Genom. Bioinform 2, lqaa078. 10.1093/nargab/lqaa078. 33015620 PMC7518324

[55] Behdenna A, Colange M, Haziza J, Gema A, Appé G, Azencott CA, and Nordor A. (2023). pyComBat, a Python tool for batch effects correction in high-throughput molecular data using empirical Bayes methods. BMC Bioinf. 24, 459. 10.1186/s12859-023-05578-5. PMC10701943 38057718

[56] Tarasov A, Vilella AJ, Cuppen E, Nijman IJ, and Prins P (2015). Sambamba: fast processing of NGS alignment formats. Bioinformatics 31, 2032–2034. 10.1093/bioinformatics/btv098. 25697820 PMC4765878

[57] Koboldt DC, Zhang Q, Larson DE, Shen D, McLellan MD, Lin L, Miller CA, Mardis ER, Ding L, and Wilson RK (2012). VarScan 2: somatic mutation and copy number alteration discovery in cancer by exome sequencing. Genome Res. 22, 568–576. 10.1101/gr.129684.111. 22300766 PMC3290792

[58] Sondka Z, Dhir NB, Carvalho-Silva D, Jupe S, Madhumita McLaren K., Starkey M, Ward S, Wilding J, Ahmed M, (2024). COSMIC: a curated database of somatic variants and clinical data for cancer. Nucleic. Acids Res 52, D1210–D1217. 10.1093/nar/gkad986. 38183204 PMC10767972

[59] Garman B, Anastopoulos IN, Krepler C, Brafford P, Sproesser K, Jiang Y, Wubbenhorst B, Amaravadi R, Bennett J, Beqiri M, (2017). Genetic and Genomic Characterization of 462 Melanoma Patient-Derived Xenografts, Tumor Biopsies, and Cell Lines. Cell Rep. 21, 1936–1952. 10.1016/j.celrep.2017.10.052. 29141224 PMC5709812

[60] Xiao M, Rebecca VW, and Herlyn M (2019). A Melanoma Patient-Derived Xenograft Model. J. Vis. Exp 147, e59508. 10.3791/59508. PMC6986799 31157772

[61] Shehwana H, Kumar SV, Melott JM, Rohrdanz MA, Wakefield C, Ju Z, Siwak DR, Lu Y, Broom BM, Weinstein JN, (2022). RPPA SPACE: an R package for normalization and quantitation of Reverse-Phase Protein Array data. Bioinformatics 38, 5131–5133. 10.1093/bioinformatics/btac665. 36205581 PMC9665860

[62] Akbani R, Ng PKS, Werner HMJ, Shahmoradgoli M, Zhang F, Ju Z, Liu W, Yang JY, Yoshihara K, Li J, (2014). A pan-cancer proteomic perspective on The Cancer Genome Atlas. Nat. Commun 5, 3887. 10.1038/ncomms4887. 24871328 PMC4109726

[63] Li H, and Durbin R (2009). Fast and accurate short read alignment with Burrows-Wheeler transform. Bioinformatics 25, 1754–1760. 10.1093/bioinformatics/btp324. 19451168 PMC2705234

[64] Cingolani P, Platts A, Wang LL, Coon M, Nguyen T, Wang L, Land SJ, Lu X, and Ruden DM (2012). A program for annotating and predicting the effects of single nucleotide polymorphisms, SnpEff: SNPs in the genome of Drosophila melanogaster strain w1118; iso-2; iso-3. Fly (Austin) 6, 80–92. 10.4161/fly.19695. 22728672 PMC3679285

[65] Bushnell B (2014). BBMap: A Fast, Accurate, Splice-Aware Aligner (Ernest Orlando Lawrence Berkeley National Laboratory).

[66] Langmead B, Trapnell C, Pop M, and Salzberg SL (2009). Ultrafast and memory-efficient alignment of short DNA sequences to the human genome. Genome Biol. 10, R25. 10.1186/gb-2009-10-3-r25. 19261174 PMC2690996

[67] Steele CD, Abbasi A, Islam SMA, Bowes AL, Khandekar A, Haase K, Hames-Fathi S, Ajayi D, Verfaillie A, Dhami P, (2022). Signatures of copy number alterations in human cancer. Nature 606, 984–991. 10.1038/s41586-022-04738-6. 35705804 PMC9242861

[68] Hicks SC, Okrah K, Paulson JN, Quackenbush J, Irizarry RA, and Bravo HC (2018). Smooth quantile normalization. Biostatistics 19, 185–198. 10.1093/biostatistics/kxx028. 29036413 PMC5862355

[69] Zhao Y, Wong L, and Goh WWB (2020). How to do quantile normalization correctly for gene expression data analyses. Sci. Rep 10, 15534. 10.1038/s41598-020-72664-6. 32968196 PMC7511327

[70] Huang KL, Mashl RJ, Wu Y, Ritter DI, Wang J, Oh C, Paczkowska M, Reynolds S, Wyczalkowski MA, Oak N, (2018). Pathogenic Germline Variants in 10,389 Adult Cancers. Cell 173, 355–370.e14. 10.1016/j.cell.2018.03.039. 29625052 PMC5949147

[71] Sollis E, Mosaku A, Abid A, Buniello A, Cerezo M, Gil L, Groza T, Güneş O, Hall P, Hayhurst J, (2023). The NHGRI-EBI GWAS Catalog: knowledgebase and deposition resource. Nucleic Acids Res. 51, D977–D985. 10.1093/nar/gkac1010. 36350656 PMC9825413

[72] Landrum MJ, Lee JM, Benson M, Brown GR, Chao C, Chitipiralla S, Gu B, Hart J, Hoffman D, Jang W, (2018). ClinVar: improving access to variant interpretations and supporting evidence. Nucleic Acids Res. 46, D1062–D1067. 10.1093/nar/gkx1153. 29165669 PMC5753237

[73] Chakravarty D, Gao J, Phillips SM, Kundra R, Zhang H, Wang J, Rudolph JE, Yaeger R, Soumerai T, Nissan MH, (2017). OncoKB: A Precision Oncology Knowledge Base. JCO Precis. Oncol. 2017. 10.1200/po.17.00011. PMC5586540 28890946

[74] Dandou S, Vendrell JA, Solassol J, Louveau B, Lebbé C, Mourah S, Rambow F, Richard E, Du Manoir S, Mangé A, (2025). MelanoDB: A dataset of clinical and molecular features of patients with advanced melanoma treated with MAPK inhibitors. Sci. Data 12, 1144. 10.1038/s41597-025-05291-3. 40615439 PMC12227540

[75] Jumper J, Evans R, Pritzel A, Green T, Figurnov M, Ronneberger O, Tunyasuvunakool K, Bates R, Žídek A, Potapenko A, (2021). Highly accurate protein structure prediction with AlphaFold. Nature 596, 583–589. 10.1038/s41586-021-03819-2. 34265844 PMC8371605

[76] Varadi M, Anyango S, Deshpande M, Nair S, Natassia C, Yordanova G, Yuan D, Stroe O, Wood G, Laydon A, (2022). Alpha-Fold Protein Structure Database: massively expanding the structural coverage of protein-sequence space with high-accuracy models. Nucleic Acids Res. 50, D439–D444. 10.1093/nar/gkab1061. 34791371 PMC8728224

[77] Van Allen EM, Wagle N, Sucker A, Treacy DJ, Johannessen CM, Goetz EM, Place CS, Taylor-Weiner A, Whittaker S, Kryukov GV, (2014). The genetic landscape of clinical resistance to RAF inhibition in metastatic melanoma. Cancer Discov. 4, 94–109. 10.1158/2159-8290.Cd-13-0617. 24265153 PMC3947264

