# Supplementary material for: Melanoma-patient-derived xenograft multi-omics resource melPDomiX maps gain- and loss-of-function alterations
Source: Cell Rep. Author manuscript; Available in PMC 2026 Aug 13. (PMC13470634; doi:10.1016/j.celrep.2026.117455)
Supplement: Supplementary file 1 [file NIHMS2190728-supplement-1.pdf]

Cell Reports, Volume *45*

Supplemental information

Melanoma-patient-derived xenograft multi-omics resource melPDomiX maps gain- and loss-of-function alterations

Konstantinos Tsingas, Monzy Thomas, McKenna Reale, Min Xiao, Jayamanna Wickramasinghe, Yeqing Chen, Bryant Duong, Anastasia Lucas, Gatha Thacker, Haiyin Li, Haiwei Mou, Eric Ramirez-Salazar, Abdurrahman Elbasir, Jessie Villanueva, Xiaowei Xu, Ahron Flowers, Giorgos C. Karakousis, John T. Miura, Tara C. Mitchell, Ravi K. Amaravadi, Lynn M. Schuchter, Shujing Liu, Qi Long, Dave S.B. Hoon, Romela I. Ramos, Jose J. Catbagan, Matias A. Bustos, Jeffrey E. Gershenwald, Julie M. Simon, Jennifer A. Wargo, Michael A. Davies, Yiling Lu, Gordon B. Mills, Sonia Cohen, Aleigha Lawless, Tatyana Sharova, Dennie T. Frederick, Keith T. Flaherty, Nir Hacohen, Genevieve M. Boland, Noam Auslander, and Meenhard Herlyn

Supplementary Tables

Table S1. Clinical characteristics of melPDomiX patients

Table S2. Whole Exome sequencing dataset of melPDomiX called mutations without matched normal using samtools, by gene and variant.

Table S3. Whole Exome sequencing dataset of melPDomiX called mutations with matched normal using VARSCAN2, by gene and variant.

Table S4. Literature-based GOF and LOF variants in melanoma.

Table S5. Double substitutions within the same amino acid occurring in multiple PDX through this cohort. The melPDomiX sample count for each observed substitution is provided.

Table S6. RNA sequencing dataset (normalized values) of melPDomiX by gene name or ensemble ID.

Table S7. RPPA dataset (normalized values) of melPDomiX by gene name and antibody ID.

Table S8. Variant categorization into expression gain or loss, based on observed increase or decrease in gene expression in variant-positive samples, with agreement through the PDX and TCGA SKCM cohort. Each assigned gain or loss of expression variant effect size is also provided with a germline or somatic categorization of based on the melPDomiX and TCGA SKCM cohorts, using VARSCAN2 called mutations.

Table S9. Variant categorization into GOF/LOF or GOE/LOE status by MOFA and the structural context representation.

Table S10. Copy number alterations of the melPDomiX cohort

Table S11. Pathway information with the respective gene set for each enriched pathway identified

Table S12. Cross reference analysis of selected variants with SNP databases, reporting RSID and associations identified across different databases investigated.

Supplementary Figures

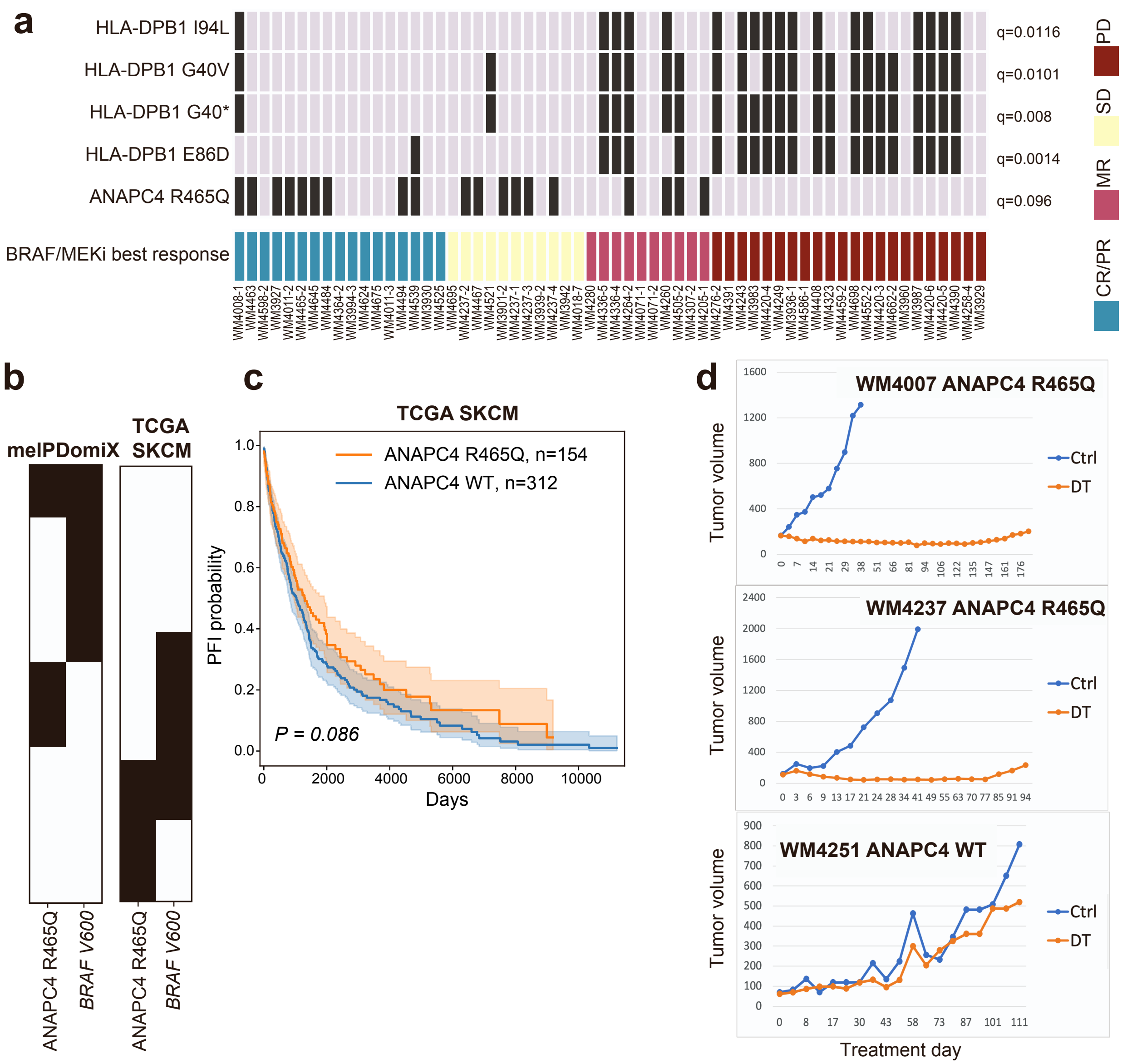

Figure S1. Characterization of ANAPC4 and HLA-DPB1 mutations with response-specific melanoma patterns

(a) *ANAPC4* R465Q status is uniquely observed in PDX with clinical benefit from BRAF/MEKi (CR/PR, SD and MR), whereas *HLA-DPB1* mutations G40V/*, I94L and E86D are almost uniquely observed in patients with no clinical benefit, namely PD or those with MR. CR/PR - Complete/Partial Response, SD - Stable Disease, MR – Mixed Response, PD – Progressive Disease. FDR corrected Fisher exact test q-values are reported for each variant, testing the enrichment between highlighted groups of treatment. **(b)** A subset of *BRAF* V600E/K mutant tumors are positive for *ANAPC4* R465Q mutations in both the melPDomiX and TCGA SKCM cohorts. **(c)** TCGA SKCM tumors that are positive for *ANAPC4* R465Q status in germline have slightly improved progression free interval (log-rank p-value = 0.86). **(d)** Three melanoma tumors, two harboring the *ANAPC4* R465Q mutation (WM4007 and WM4237) and one *ANAPC4* wild-type tumor (WM425), were implanted into NSG mice. Mice were treated with either

Dabrafenib/Trametinib or vehicle control. Tumor growth was monitored longitudinally at multiple time points to assess differential responsiveness to BRAF/MEK inhibition over time.

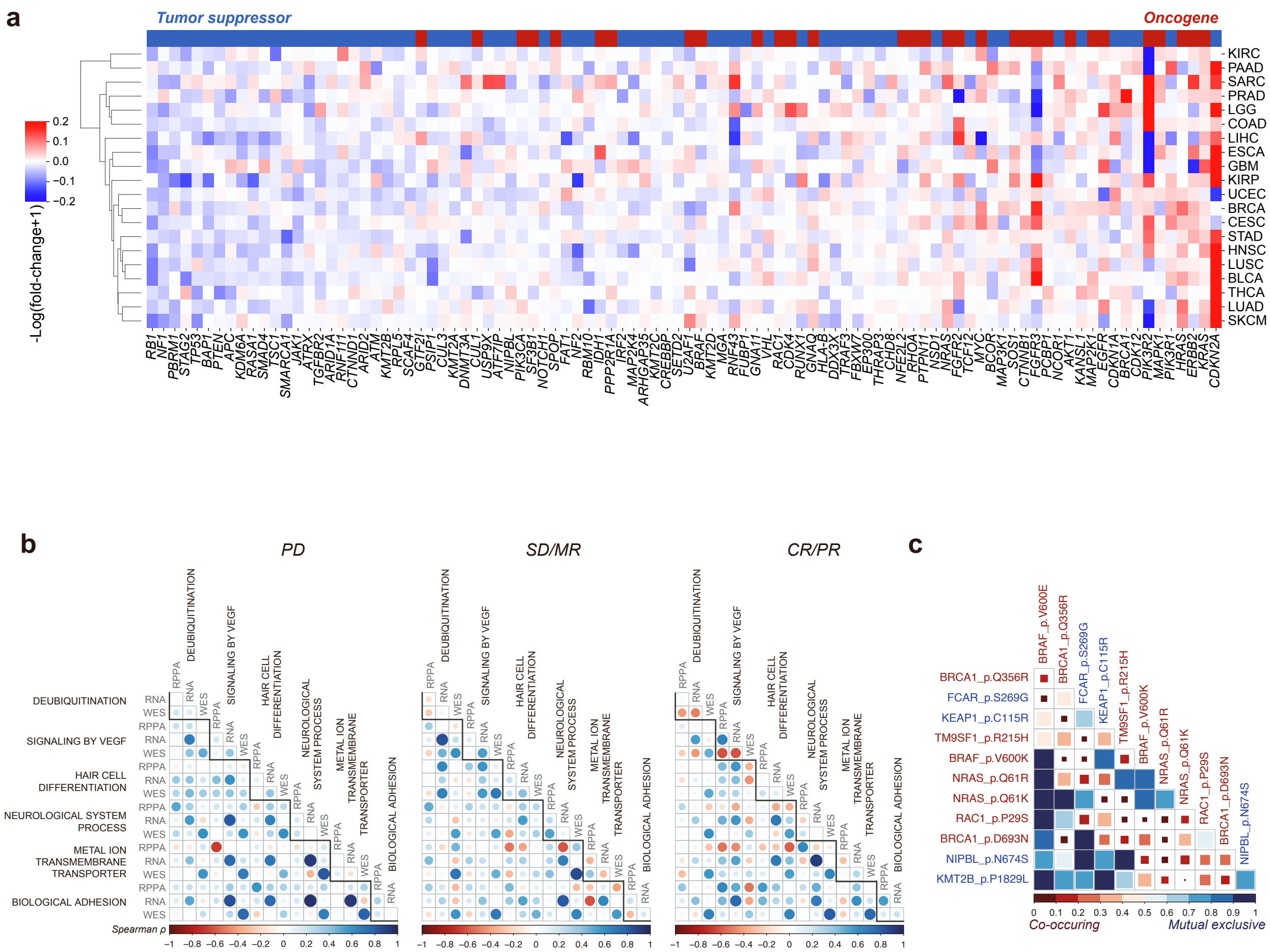

Figure S2. Mutation-associated expression shifts, response-specific pathway correlations across omics, and variant co-occurrence patterns

(a) Fold-change (-log +1 scaled) analysis comparing gene expression in mutated (missense mutations only) versus wild-type samples for driver genes annotated as pan-cancer tumor suppressors (blue) or oncogenes (red).

(b) Correlation plots correlating pathway scores calculated using each omics type, for PDX from patients demonstrating progressive disease (PD, left panel), stable disease/mixed response (SD/MR, middle panel), and complete/partial response (CR/PR, right panel) to BRAF/MEK inhibitor therapy. **(c)** Mutual exclusivity and co-occurrence patterns of potential GOE and LOE melanoma variants.

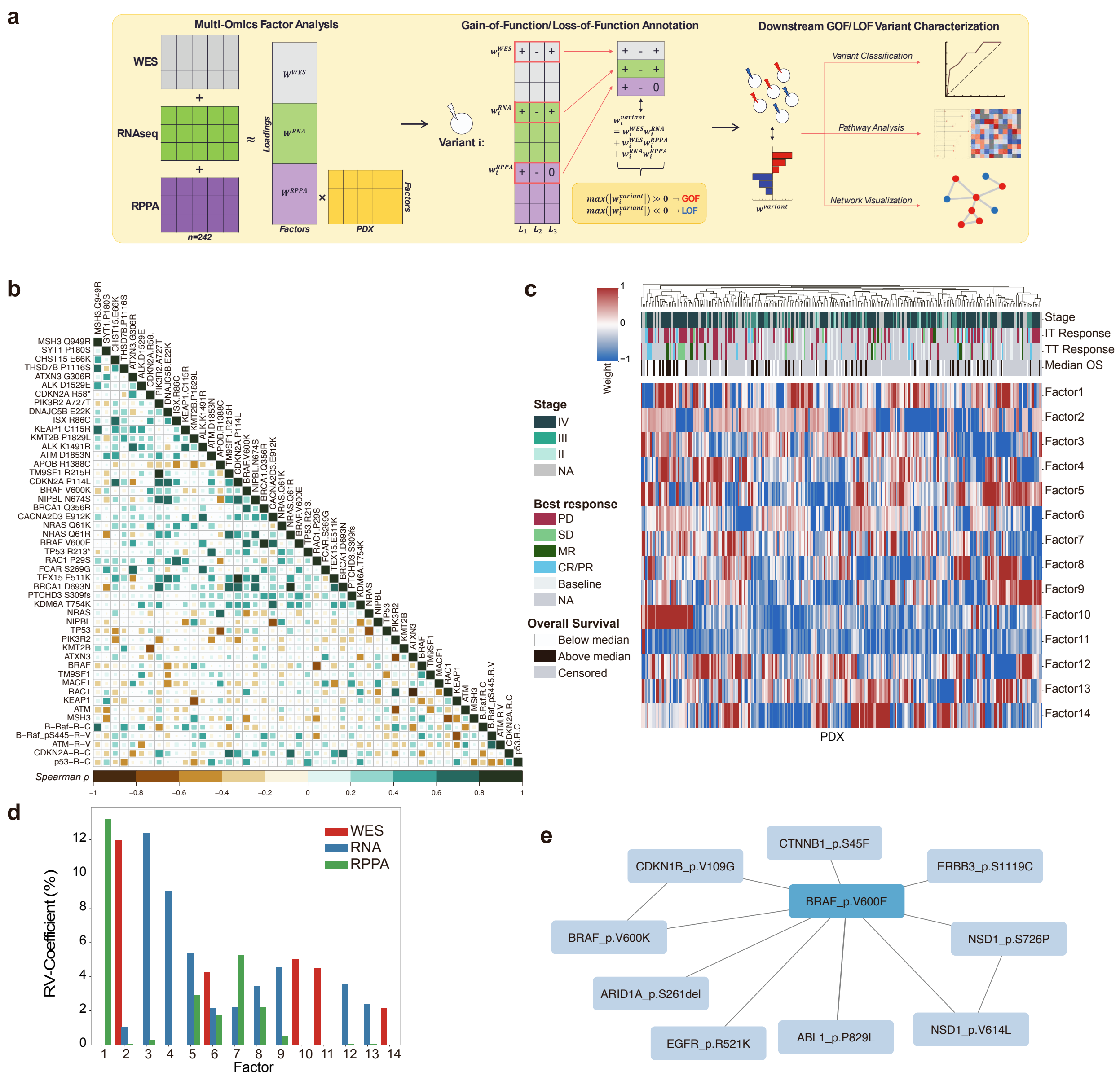

Figure S3. Multi-omics factor analysis reveals consistent GOF vs LOF concordance.

(a) Schematic overview of the computational pipeline using MOFA multi-omics integration to distinguish GOF/GOE vs LOF/LOE variants. **(b)** Correlation plot showing the Spearman correlation coefficients of factor loadings across variant, gene and proteins of GOF/GOE and LOF/LOE annotated variants through the multi-omics factor analysis. **(c)** Heatmap showing the factor loadings (rows) for each PDX with the respective clinical information. **(d)** Bar plot showing the variation explained per modality across factors. **(e)** Network of variants that are connected via consistent GOF/LOF concordance across >50% of latent factors.

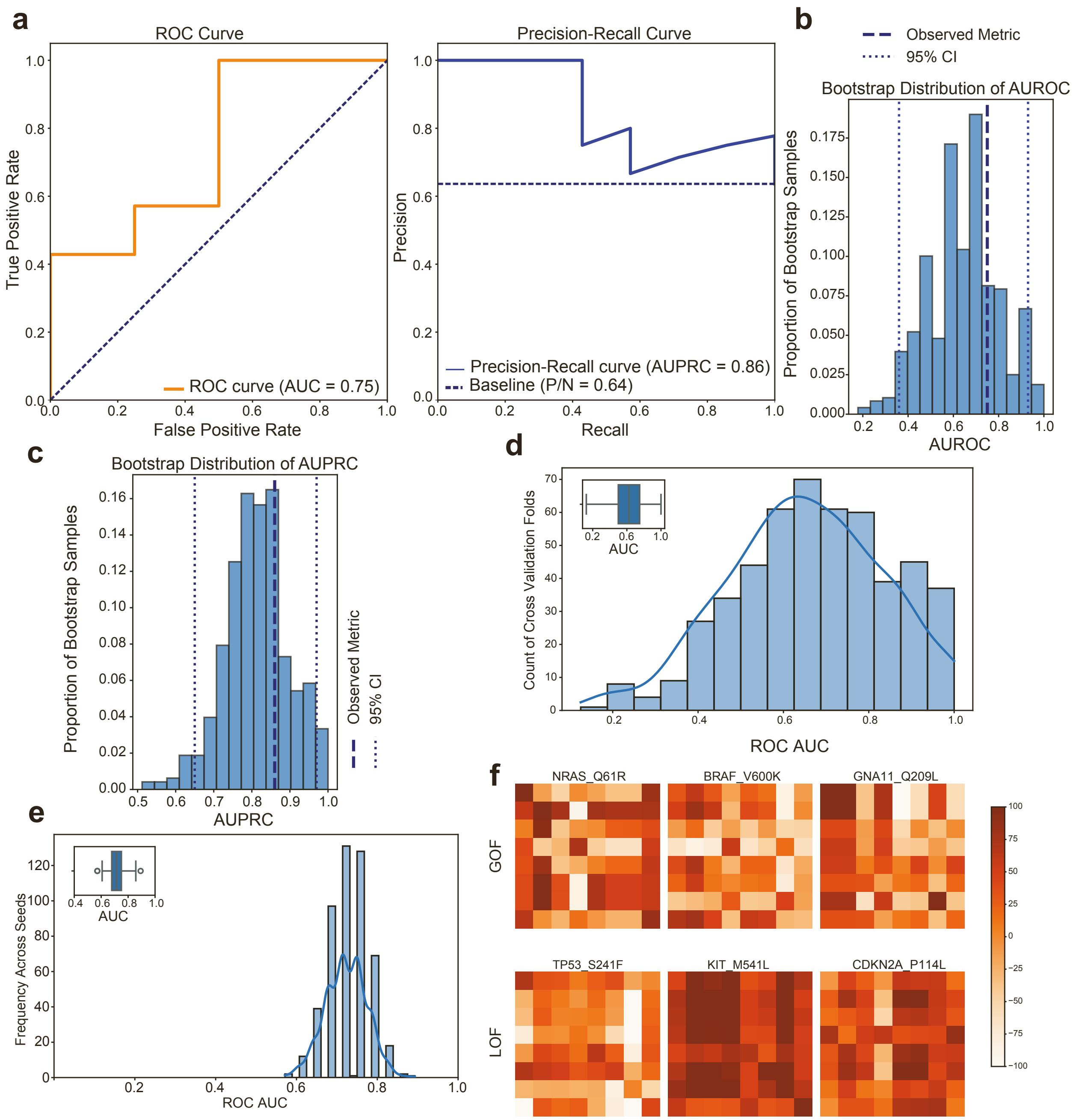

Figure S4. Multi-omics factor analysis and alphamissense representations classify GOF vs LOF variants

(a) Receiver operating characteristic (ROC) and precision recall curves (AUC = 0.75, AUPRC = .86) for predicting GOF and LOF variants (n=11) in the multi-omics factor

analysis. **(b),(c)** AURPC and AUPRC distributions across 500 bootstrapping samples when classifying literature reported GOF vs. LOF variants with MOFA variant scoring. **(d)** ROC AUC distributions (Mean AUC = 0.64) for the RandomForestClassifier on the 8x8x4 training set (n=31) using a 75-25 split across 500 cross validation folds. **(e)** Model score distributions (Mean AUC = 0.73) for models built using 8x8x4 AMS sequence representation tested on the melanoma GOF/LOF variants verified by literature. **(f)** AMS Logit values along the first and second dimension of the 8x8x4 AlphaMissense representation for verified literature GOF/LOF associated variants.

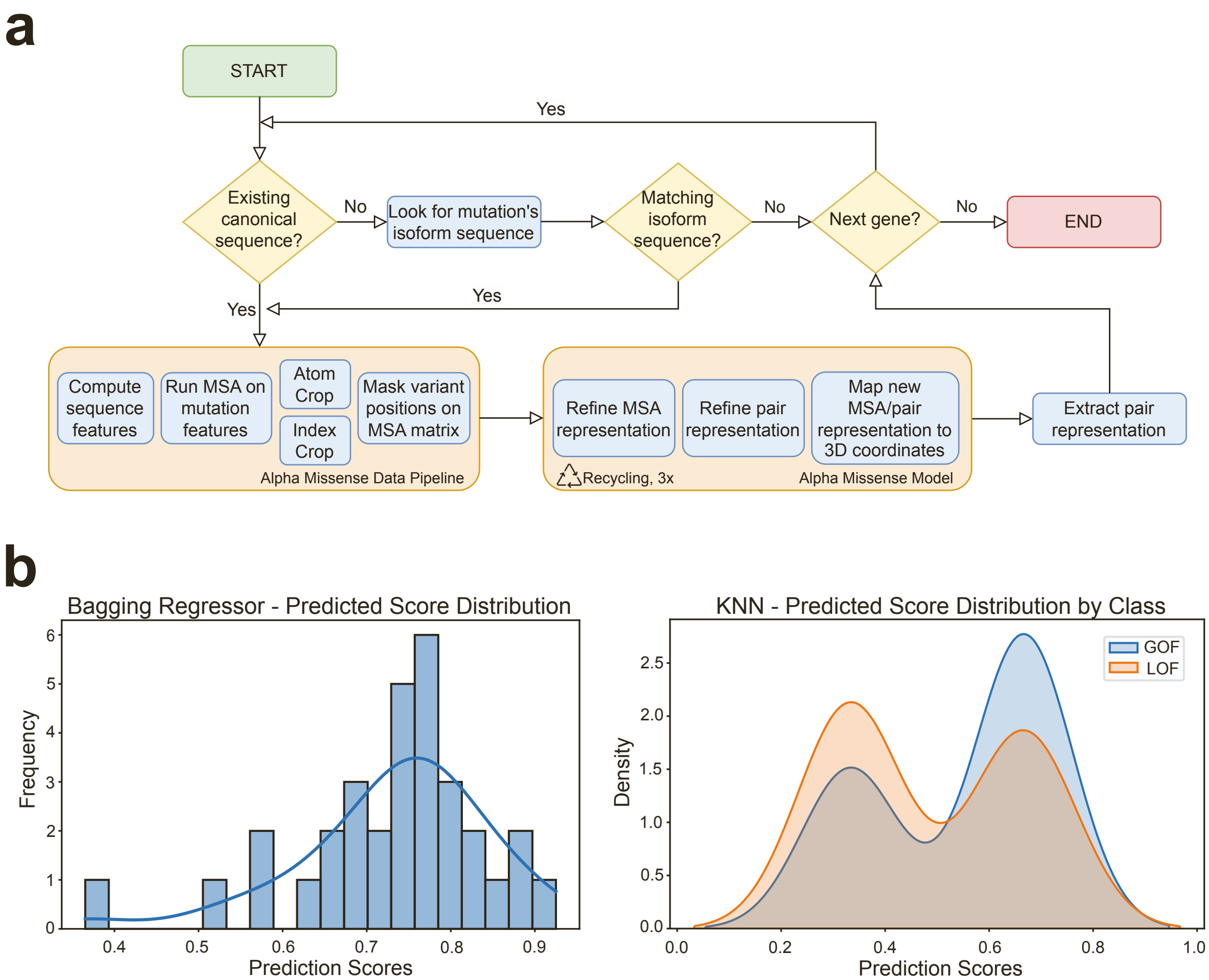

Figure S5. Framework for AlphaMissense variant context representation trained to predict GOF vs. LOF.

(a) Flowchart describing the machine learning framework to predict GOF vs LOF melanoma variants using AlphaMissense (AMS) sequence representation. **(b)** Model score distributions for models built using AMS sequence representation when applying a bagging repressor (left panel) and KNN (right panel).

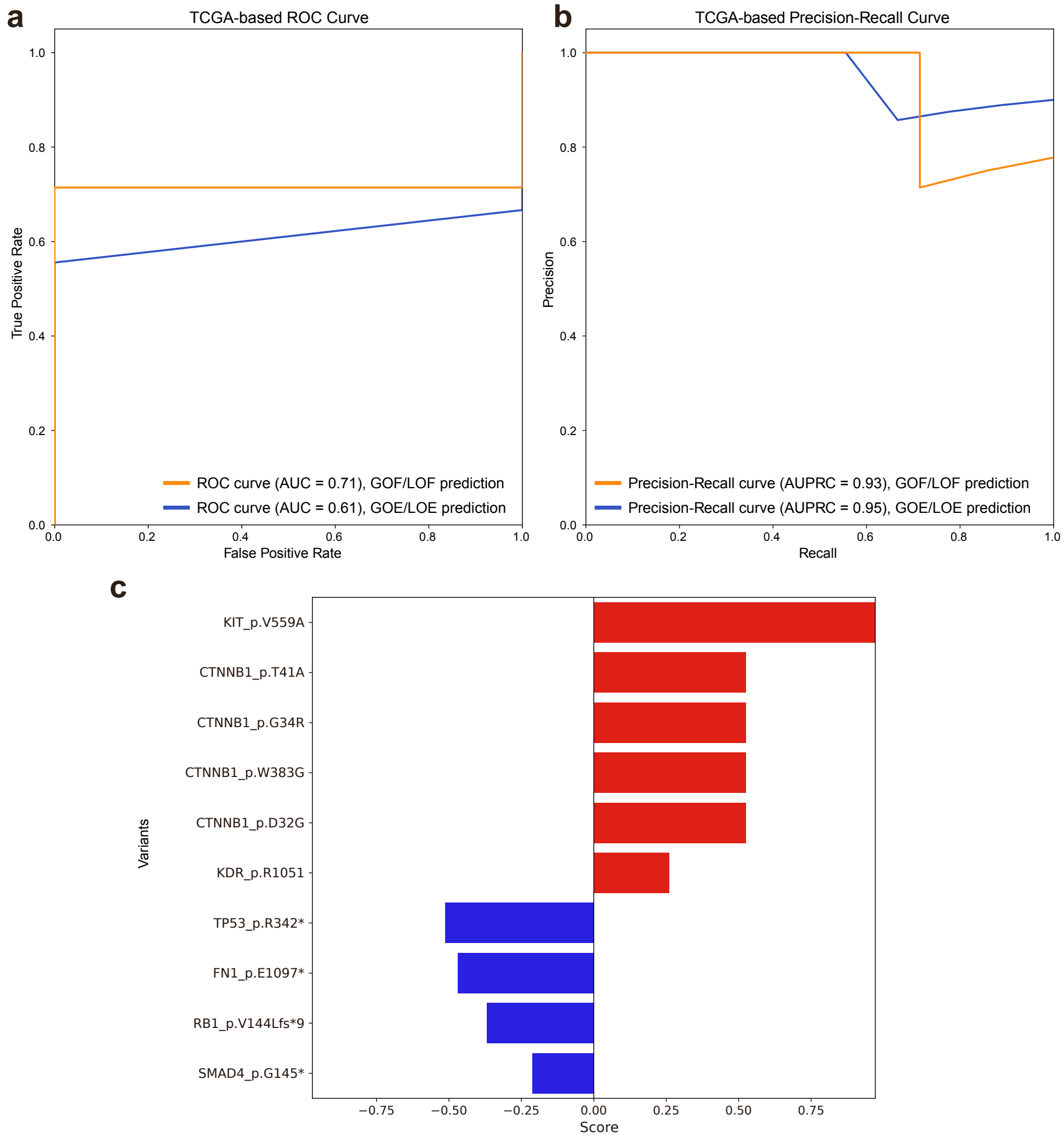

Figure S6. Multi-omics factor analysis framework predicts GOF vs LOF variants in TCGA SKCM cohort.
(a),(b) Receiver operating characteristic (ROC, a) and precision recall curves (b) for predicting GOF vs. LOF variants (orange) and GOE vs. LOE variants (blue) using multi-omics factor analysis when applied to TCGA SKCM data. **(c)** Barplot showing selected highly ranked predicted GOF/GOE and LOF/LOE variants using the TCGA SKCM MOFA-based predictor, applied to all variants.

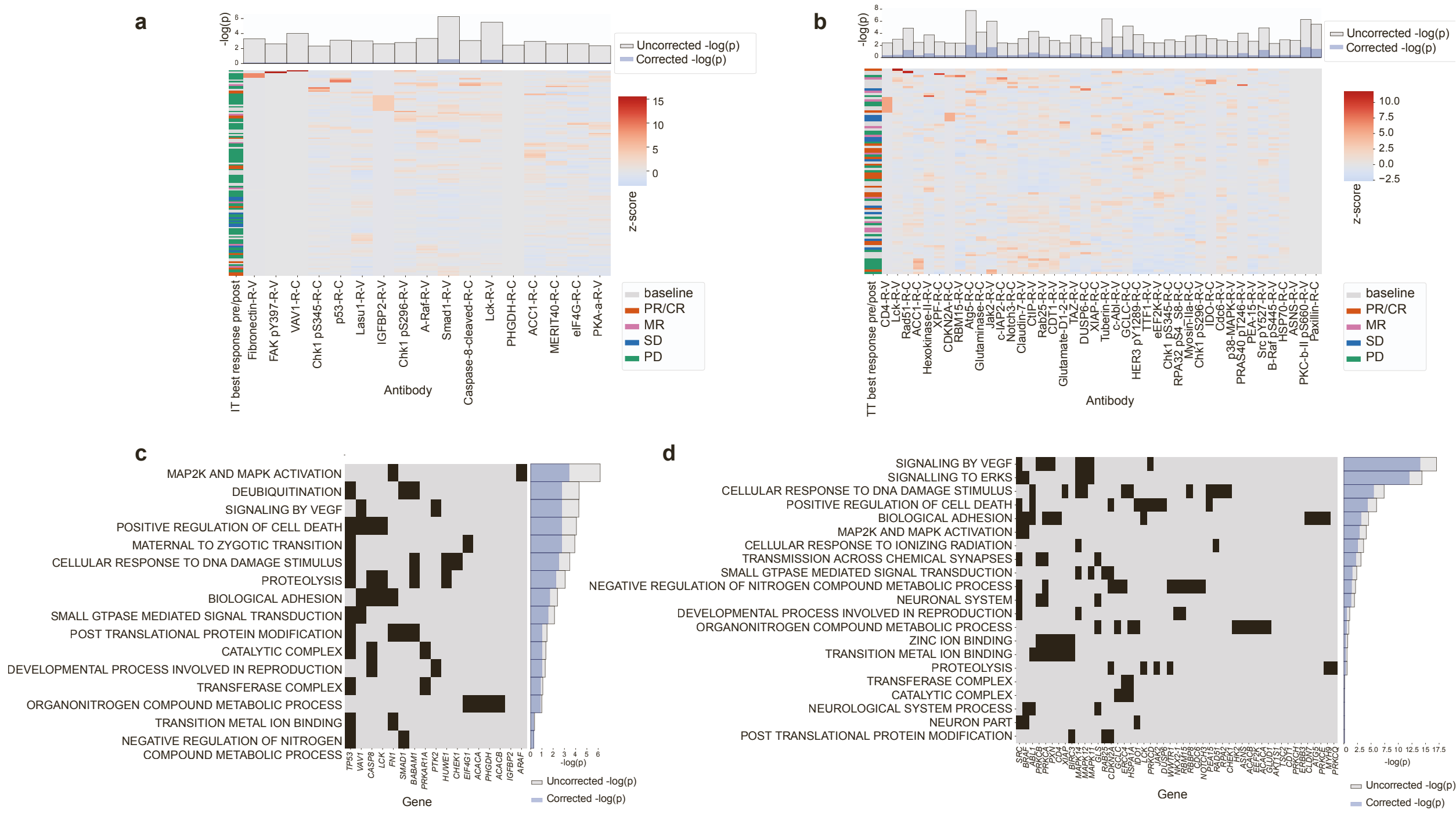

Figure S7. RPPA abundance and pathway enrichment analysis across immunotherapy and target therapy groups.

(a),(b) Heatmaps of RPPA normalized abundance (z-score) with overlaid uncorrected and FDR-corrected p-values, evaluating RPPA antibody association with treatment response, for immunotherapy (a, anti-PD1) and (b) targeted therapy (b, BRAFi/MEKi). Mann-Whitney-U p-values are reported, comparing between patients demonstrating progressive disease and complete/partial response (using a selection cutoff of uncorrected p-value < 0.1). **(c),(d)** Pathway enrichment analysis of genes representing proteins from (a),(b), respectively. Uncorrected and FDR-corrected p-values of hypergeometric enrichment testing for each pathway are shown.

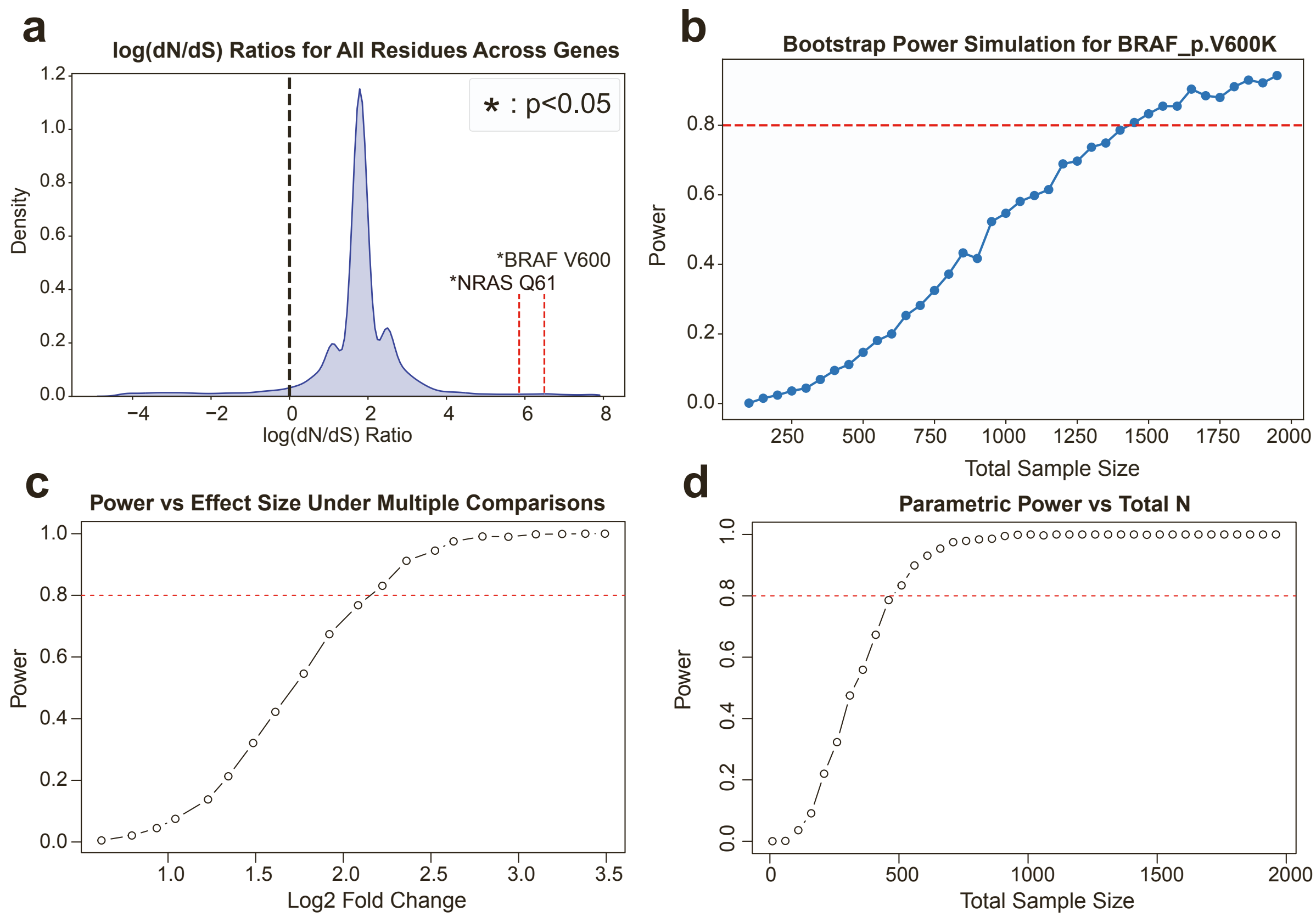

Figure S8. dN/dS distribution and power analyses across sample size and effect size

(a) Histogram showing the log(dN/dS) ratios for all residues across genes. **(b)** Power (y-axis) vs total sample size (x-axis) for bootstrap simulation of BRAF V600K mutations. **(c)** Power (y-axis) vs log2 fold change under multiple comparisons. **(d)** Parametric power (y-axis) vs total sample size (x-axis) evaluation.
